# Effect of smoking cessation medications on intracranial aneurysm risk: A Mendelian randomization study

**DOI:** 10.18332/tid/186171

**Published:** 2024-04-30

**Authors:** Xin Liang, Xin Tong, Yan Miao, Xiaopeng Xue, Aihua Liu, Feng Guan

**Affiliations:** 1 Department of Neurosurgery, Beijing Shijitan Hospital, Capital Medical University, Beijing, China; 2 Beijing Neurosurgical Institute, Beijing Tiantan Hospital, Capital Medical University, Beijing, China; 3 Department of Neurosurgery, The Third Xiangya Hospital, Central South University, Changsha, China

**Keywords:** aneurysm, genetics, smoking, smoking cessation, Mendelian randomization

## Abstract

**INTRODUCTION:**

We aim to assess the association between smoking behavior and intracranial aneurysms (IAs) and the effect of smoking cessation medications on IAs at the genetic level.

**METHODS:**

Causal effects of four phenotypes: 1) age at initiation of regular smoking, 2) cigarettes smoked per day, 3) smoking cessation, and 4) smoking initiation on IAs, were analyzed using two-sample inverse-variance weighted Mendelian randomization analyses. The effects of genes interacting with the smoking cessation medications were analyzed using cis-expression quantitative trait loci genetic instruments on IAs using summary statistics-based Mendelian randomization analyses. Colocalization analyses were then used to test whether the genes shared causal variants with IAs. The role of confounding phenotypes as potential causative mechanisms of IAs at these gene loci was tested.

**RESULTS:**

Cigarettes smoked per day (OR=2.89; 95% CI:1.85–4.51) and smoking initiation on IAs (OR=4.64; 95% CI: 2.64–8.15) were significantly associated with IA risk. However, age at initiation of regular smoking (OR=0.54; 95% CI: 0.10–2.8) and smoking cessation (OR=6.80; 95% CI: 0.01–4812) had no overall effect on IAs. Of 88 genes that interacted with smoking cessation medications, two had a causal effect on IA risk. Genetic variants affecting HYKK levels showed strong evidence of colocalization with IA risk. Higher HYKK levels in the blood were associated with a lower IA risk. Gene target analyses revealed that cigarettes/day could be a main mediator of HYKK’s effect on IA risk.

**CONCLUSIONS:**

This study provides evidence supporting that smoking initiation on IAs and cigarettes/day may increase IA risk. Increased HYKK gene expression may reduce IA risk. This can be explained by the increased number of cigarettes consumed daily. HYKK could also reduce IA risk due to the positive effect of continuous abstinence and varenicline therapy on smoking cessation.

## INTRODUCTION

The prevalence of unruptured intracranial aneurysms (uIAs) in the general population is approximately 3%^1^. Intracranial aneurysm (IA) rupture is the leading cause of subarachnoid hemorrhage (SAH), with a case fatality rate of approximately 35%^2^. Although the pathophysiology remains unclear, previous studies have identified smoking as one of the most important risk factors for IA, affecting IA generation, growth, rupture, and even treatment prognosis^3-5^. One previous study also found that the global incidence of SAH is declining as smoking prevalence decreases^6^. Therefore, most neurosurgeons suggest that patients with IA should be counseled on the importance of smoking cessation^7,8^. However, smoking cessation is challenging because it requires individuals to overcome physical nicotine dependence and long-standing rewarding behaviors. A previous study reported that although approximately 70% of people who smoked cigarettes wanted to quit smoking, only 7.5% remained abstinent for one year^9^.

Pharmacotherapy has been identified as either first- or second-line treatment to assist people who smoke in quitting attempts^10^. The effectiveness of these medications for smoking cessation is well established^11,12^. In the secondary prevention of stroke, pharmacotherapy with counseling was associated with more cost-effectiveness than brief counseling alone^13^. Moreover, the number of incidentally discovered IAs is increasing due to imaging detection technologies’ increasing availability and quality^5^. Using smoking cessation medications to increase the rate of smoking cessation in patients who want to quit smoking seems to have great potential for secondary prevention of IA and SAH. However, it is still unclear whether the administration of smoking cessation medications affects the risk of IA or SAH.

The increase in summary data from genome-wide association studies (GWAS) has promoted the maturation of Mendelian randomization (MR) analysis, which has mainly been applied to estimate the relationship between exposures and outcomes^14^. The largest previous GWAS reported that drug target enrichment showed pleiotropy between IA and smoking cessation medications^15^. Understanding this pleiotropy and its mechanisms will promote further understanding of the mechanisms of IA development. Additionally, it may help identify the effect of smoking cessation medications on IA risk. Therefore, this study aimed to gain insights into the association between smoking cessation medications and IA, using a MR analysis.

## METHODS

### Study design and data sets

**Figure 1:**
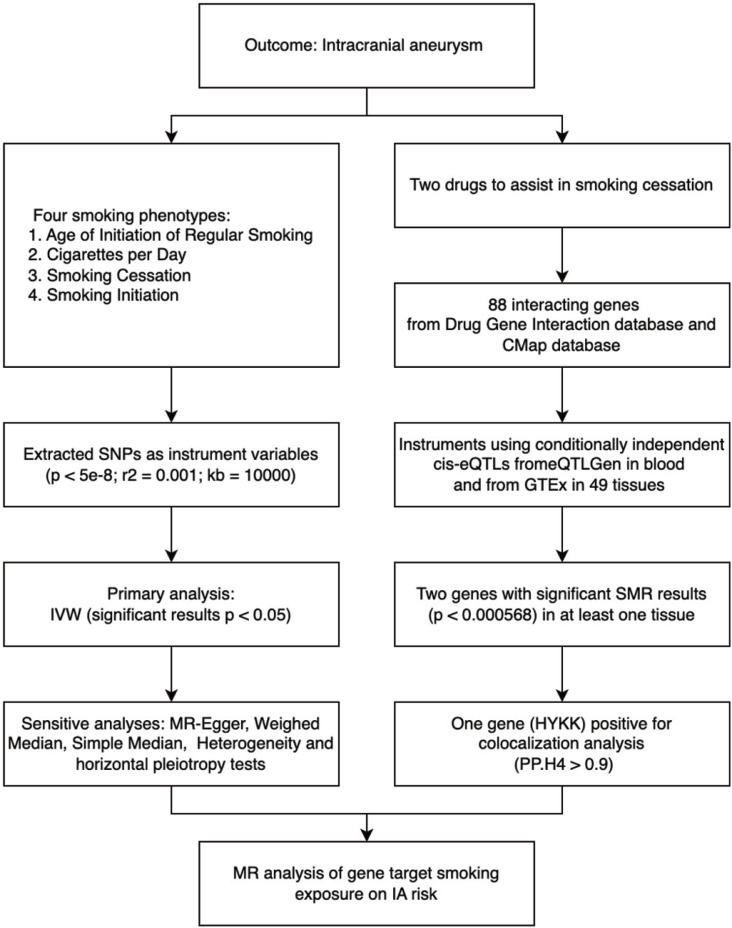
Diagram of the study design

This study used publicly available de-identified summary data from previous studies. Ethical approval was obtained in all the original studies. The study design is illustrated in Figure 1. Generally, MR was used to test: 1) the effect of different smoking exposure phenotypes on IA risk, and 2) the effects of gene targets that interacted with smoking cessation medications on IA.

The largest GWAS dataset on IAs in a previous study was used as the primary outcome for both analyses^15^. This dataset included 7495 cases and 71934 controls of European ancestry. The second outcome was unruptured and ruptured IA in the same study^15^. In Analysis 1, four smoking phenotypes from a larger meta-analysis were used^16^. Age at initiation of regular smoking (n=341427) was defined as the age at which an individual began smoking cigarettes regularly. Cigarettes smoked per day (n=337334) were defined as the average number of cigarettes smoked per day. Smoking cessation (n=547219) was a binary phenotype compared between current and former smokers. Smoking initiation on IAs (n=1232091) was also a binary phenotype that compared any participant reporting ever being a regular smoker with those who reported never being a regular smoker. In Analysis 2, the available expression quantitative trait loci (eQTLs) for drug target genes were used as proxies for exposure to each smoking cessation medication. eQTLs summary-level data were obtained from the eQTLGen Consortium^17^ and GTEx Consortium V8^18^. Only cis-eQTLs were included in the present study; they were defined as eQTLs within 1 megabase (1 Mb) on either side of the encoded gene.

### Selection of genes that interacted with smoking cessation medication

The current study included two primary smoking cessation medications: varenicline and bupropion. Gene targets were selected for these two drugs using the drug-gene interaction database^19^ and connectivity map resource^20^. In the connectivity map, genes for which overexpression or knockdown resulted in gene expression patterns with a connectivity score >90% or < -90% compared to gene expression patterns were selected in response to the administration of either drug of interest. If >20 genes passed this threshold, the 20 most similar or dissimilar genes were selected.

### Selection of genetic instruments

Single nucleotide polymorphisms (SNPs) associated with each smoking phenotype at a genome-wide significance level were selected in Analysis 1 (p<5×10^-8^). The independence of the SNPs was assessed using stringent criteria (LD r2, 0.001; clumping window, 10000 kb). In Analysis 2, common (MAF>1%) eQTLs SNPs significantly (p<5.0×10^-8^) associated with the expression of genes associated with smoking cessation medication in each tissue were identified, and genes with no eQTLs in the blood or other tissues available at a significance level were excluded.

### Mendelian randomization

MR analyses were conducted using the R package Two Sample MR. The inverse variance weighted (IVW) MR method was selected as the primary MR analysis method in Analysis 1. Statistical significance was set at p<0.05. Summary-data-based MR (SMR) was selected as the primary method in Analysis 2. Statistical significance was set at p<5.68×10^-4^ (0.05 Bonferroni-corrected for 88 genes). SMR investigates the association between gene expression levels and the outcome of interest using summary-level data from GWAS and eQTL studies^21^. Allele harmonization and analysis were performed using SMR software version 1.03. All tests conducted were two-tailed.

### Sensitivity analyses

Several sensitivity analyses were performed to determine the presence of heterogeneity and pleiotropy in genetic variants. For the IVW, MR-Egger, weighted median, weighted mode, and simple mode analyses were also performed. Similar beta effect directions were considered significant for these tests. Cochran’s Q test was used to assess heterogeneity. MR-Egger regression and MR Pleiotropy RESidual Sum and Outlier tests were used to assess the potential horizontal pleiotropy of the SNPs used as instrument variants. All sensitivity analyses were considered statistically significant at p<0.05. For SMR, heterogeneity in dependent instruments test was used to determine whether the observed association between gene expression and the outcome was due to a linkage scenario. This analysis was performed using the SMR software. The heterogeneity in the dependent instruments test showed p<0.05, indicating that the association was probably due to linkage. All tests conducted were two-tailed tests.

### Colocalization analysis

Colocalization analysis was performed to confirm that eQTLs with putative causal effects on IA identified by SMR (defined as p<5.68×10^-4^; 0.05 Bonferroni-corrected for 88 target genes) share a causal genetic variant, using the R package Coloc (version 5.2.1) with default priors (probability of shared causal variant for trait 1 and trait 2 was P1=P2=1×10^-4^, probability of shared causal variant across two traits was P12=1×10^-5^). SNPs in the ±100 kb gene variant were included. The 1000 Genomes v3 European ancestry dataset was used as an LD reference panel. Statistically significant MR hits with posterior probability for hypothesis 4 (PP.H4) >0.9 (the probability of a shared causal variant) for at least one instrumental variant were then investigated further using the following analyses.

### MR analysis of gene target phenotype on IA risk

After selection by SMR and colocalization analysis, PubMed and PhenoScanner were searched for the phenotypes associated with the selected genes to evaluate whether potential confounder phenotypes were associated with the selected gene and IAs. The association between IA and confounding phenotypes was tested using colocalization analysis. First, phenotypes that shared causal variants in the selected gene locus with IA (PP.H4 >0.9) were selected. Then, the association between the eQTL of selected genes and confounder phenotypes was tested using SMR analysis, and the association between the confounder phenotypes and IA was tested using IVW analysis.

Finally, we tested whether the effect of the selected gene expression on IA could be explained by confounding phenotypes. If this is true, then the MR effect size estimate of confounder phenotypes genetically proxied by only the most similar shared causal variant in the colocalization analysis (exposure) on IA (outcome) is similar to the MR effect size estimate of confounder phenotypes genetically proxied by all other genetics, excluding the selected genes^22^.

## RESULTS

### Effect of smoking behavior on IA risk

**Figure 2:**
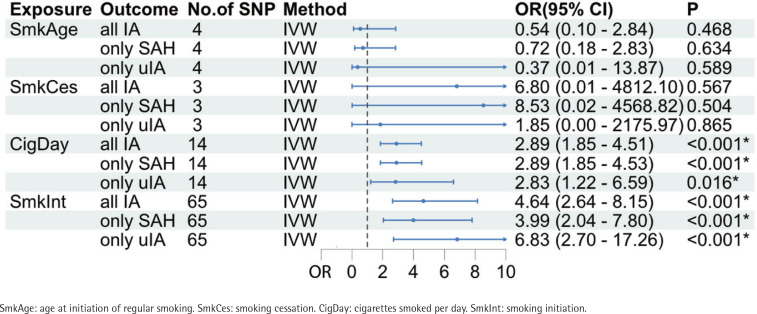
Associations of genetic predisposition to smoking phenotypes with intracranial aneurysm and its subtypes

The primary MR analysis showed cigarettes/day (OR=2.89; 95% CI: 1.85–4.51, p=2.91×10^-6^) and smoking initiation on IAs (OR=4.64; 95% CI: 2.64–8.15, p=9.62×10^-8^) were significantly associated with increased IA risk (Figure 2). However, no MR evidence of causal effects of age at initiation of regular smoking (OR=0.54; 95% CI: 0.10–2.85, p=0.468) and smoking cessation (OR=6.80; 95% CI: 0.01–4812, p=0.567) on IA risk was found. The results of patients with SAH or uIA alone were consistent with those of patients with IA (Figure 2). Significant results for cigarettes/day and smoking initiation on IAs were similar in the sensitivity analyses based on the MR-Egger, weighted median, simple mode, and weighted mode methods (Supplementary file Table S1). Cochran’s Q test indicated no evidence of heterogeneity in the MR analyses of cigarettes/day and smoking initiation on IAs for IA risk (Supplementary file Table S2). MR-Egger regression and MR Pleiotropy RESidual Sum and Outlier tests confirmed that no horizontal pleiotropy was observed in the MR analysis of cigarettes/day and smoking initiation on IAs ( Supplementary file Table S3).

### Effect of medication targets on IA risk

In total, 88 genes that interacted with smoking cessation medications were identified using the drug-gene interaction database and connectivity map (Supplementary file Table S4). Among these, 13 genes had no available eQTL data for any of the tissues in the eQTLGen or GTEx Consortium V8. After SMR analysis, two genes, including HYKK (OR=0.125; 95% CI: 0.044–0.354, p=8.74×10^-5^) in the blood and CHRNA3 (OR=0.659; 95% CI: 0.532–0.816, p=1.35×10^-4^) in the colon sigmoid, were significantly associated with IA risk (Supplementary file Table S5).

The heterogeneity in dependent instruments test suggested that the observed associations were not due to linkage (p=0.757 for HYKK and 0.082 for CHRNA3). Of these two drug targets, only HYKK showed evidence of a shared causal variant with IA (PP.H4=0.989) (Table 1). This study tested whether the effect of HYKK expression was specific to ruptured or unruptured IA. SMR showed a statistically significant effect for HYKK expression on uIA risk (OR=0.163; 95% CI: 0.334–0.784; p=0.024) (Supplementary file Table S6) and ruptured IA risk (OR=0.121; 95% CI: 0.039–0.377, p=2.69×10^-4^) in blood. These effects are consistent with the analysis of the combined IA set.

**Table 1:** Association of genetic predisposition to the HYKK gene with intracranial aneurysm and its subtypes

*Outcome*	*Tissue*	*Top SNP*	*Alleles*	*Freq*	*OR*	*95% CI*	*p of SMR*	*p of HEIDI*	*PP.H4*
All IA	Blood	*rs931794*	G/A	0.38	0.125	0.044–0.354	8.74×10^-5^	0.757	0.989
Cigarettes/day	Blood	*rs931794*	G/A	0.38	0.194	0.112–0.337	5.29×10^-9^	0.234	0.999

### Assessing the role of confounder phenotypes on IA risk in the HYKK locus

Previous studies have associated the HYKK gene with 5-hydroxylysine levels, smoking, and C-reactive protein (CRP) levels. Therefore, this study tested whether the observed effect of increased HYKK expression in the blood on IA risk could be mediated through these phenotypes (i.e. increased HYKK expression caused decreased smoking behavior, which decreased IA risk). In the colocalization analyses, only CRP levels (PP.H4=0.950) and cigarettes/day (PP.H4=0.999) showed shared causal variants with IA. HYKK also showed shared causal variants with CRP levels (PP.H4=0.986) and cigarettes/day (PP.H4=0.989) (Supplementary file Table S7). However, in the MR analysis, CRP levels lost statistical significance in the IVW analysis (p=0.257) for IA risk (Supplementary file Table S8). The association between cigarettes/day and IA risk is shown above. The direction of the causal effect of increased HYKK expression in the blood on cigarettes/day was also tested; the OR was 0.774 (95% CI: 0.684–0.877, p=5.69×10^-5^), which was in line with a previous study.

**Figure 3:**
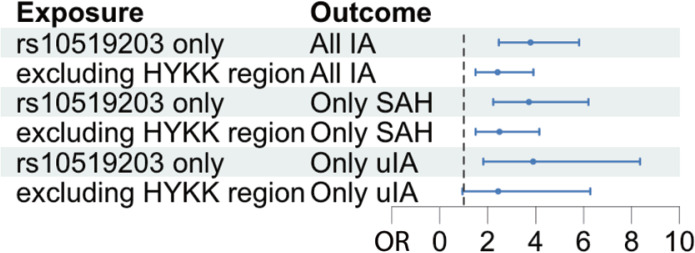
Mendelian randomization analyses to test the dependence of the HYKK locus on smoking

Finally, this study tested whether the effect of increased HYKK gene expression, genetically proxied by the most likely causal variant, *rs10519203*, on IAs could be explained mainly by cigarettes/day caused by *rs10519203*. If this is true, then the MR effect size estimate of cigarettes/day genetically proxied only by *rs10519203* (exposure) on IA (outcome) would be similar to the MR effect size estimate of cigarettes/day genetically proxied by all other genetic variants associated with cigarettes/day combined (exposure) on IA (outcome). Therefore, a Wald ratio test of cigarettes/day on IA was performed using only the variant *rs10519203* and an IVW test of GWAS associated with cigarettes/day on IA, excluding variants in the HYKK gene ±100 kb. The MR effect of cigarettes/day on IA had an OR of 3.78 (95% CI: 2.46–5.81, p=1.23×10^-9^) and 2.41 (95% CI: 1.49–3.90, p=3.41×10^-4^) using only variant *rs10519203* and variants excluding HYKK gene, respectively (Figure 3).

### Interaction type between varenicline and IA

No reports were found on the effect of varenicline administration on HYKK. Therefore, it was difficult to infer the direction of the effects of varenicline on IA risk. However, a previous study found that *rs7164594* on the HYKK locus was the most significant variant associated with continuous abstinence from 9 to 12 weeks in varenicline-treated smokers^23^. Furthermore, the current study analyzed the effects of HYKK eQTL and cigarettes/day on IA risk using only *rs7164594* as the genetic instrument. The MR analysis result showed that both HYKK eQTL (OR=0.125; 95% CI: 0.057–0.273, p=1.79×10^-7^) and cigarettes/day (OR=4.08; 95% CI: 2.13–7.83, p=2.3×10^-5^) were significantly associated with IA risk, with *rs7164594* as a genetic instrument. Similar results were observed for SAH and uIA (Supplementary file Table S9). Therefore, in addition to the direct effect of HYKK on IA risk, in smokers who were administered varenicline, the HYKK gene could also further reduce the risk of IA owing to its positive effect of continuous abstinence from smoking cessation. Based on these and the above results, the effect of HYKK on IA risk is illustrated in Supplementary file Figure 4.

## DISCUSSION

The present study showed that cigarettes/day and smoking initiation on IAs are associated with a lower risk of overall IA, SAH, and uIA. Furthermore, the study found that the pleiotropy between the genetic risk for IA and the genes interacting with smoking cessation medications established in previous studies, is partly explained by the variation in HYKK gene expression.

Smoking is one of the most established risk factors for IA in previous observation studies^3-5^. Compared to patients who never smoked, both current and former smokers had an increased IA risk or rupture risk^22,24,25^. However, although a previous study had found that a longer duration since smoking cessation was associated with decreased risk of aneurysm rupture among former smokers using univariate analysis, this association disappeared after adjustment for other confounders in the multivariable analysis^26^. This study found that smoking cessation (current smokers vs former smokers) was not associated with IA risk. This implies that, compared with current smoking, smoking cessation did not show enough effect in lowering IA risk. This phenomenon could be because changes in the arteries or other tissues resulting from smoking are irreversible to a certain extent. However, similar to previous observational studies, the present study found that the number of cigarettes/day was significantly associated with an increased risk of IA. In addition, although both former and current smokers were associated with an increased risk of IA, the effect (i.e. OR) of former smoking was often lower than that of current smoking. A previous study also found that patients who had ceased smoking had better outcomes after SAH than current smokers^27^. These results emphasize the importance of smoking cessation, especially for secondary prevention.

A targeted initiative is needed to improve smoking cessation for stroke survivors due to the unsatisfactory quitting ratio among these people^28^. One previous study found that pharmacotherapy with intensive counseling was more cost-effective than brief counseling alone for patients after a stroke^13^. Therefore, smoking cessation medications could be considered a potential approach for the first-line and secondary prevention of IA. However, the effect of smoking cessation medications on IA remains unclear. A previous study found pleiotropy between the genetic risk of IA and genes interacting with smoking cessation medications. The present study found that the HYKK gene could partly explain this association.

HYKK is located within the IA risk locus on chromosome *15q25.1* ^15^. The HYKK gene encodes hydroxylysine kinase, mainly associated with hydroxylysine kinase activity and 5-hydroxylysine level^29^. Previous studies have shown that HYKK is associated with smoking behavior and CRP levels, which are also associated with IA risk^3-5,25^. The present study found it difficult to explain the association between HYKK expression and IA risk using 5-hydroxylysine or CRP levels in the MR analyses. However, HYKK expression was also associated with cigarettes/day by SMR and colocalization analysis, which is consistent with previous studies. Colocalization analysis revealed that the most likely causal variant of IA (SNP *rs10519203*) was also associated with cigarettes/day. The effect of this variant, used as a genetic instrument, on IA risk was greater than that of genetic instruments, excluding HYKK, when cigarettes/day fully mediated the effect of these genetic instruments on IA. Considering these results, the effect of HYKK expression on the risk could be explained mainly by cigarettes/day mediation. However, a direct effect of HYKK on the risk cannot be completely ruled out.

HYKK was mainly associated with varenicline. Unfortunately, the direction of the effect of varenicline on HYKK remains unclear. Therefore, it is difficult to infer the direction of the effects of varenicline on IA risk. However, a previous study found that *rs7164594* on the HYKK locus was the most significant variant associated with continuous abstinence from weeks 9 to 12 in varenicline-treated smokers^23^. The current study found that the effects of HYKK eQTL and cigarettes/day on IA risk using only *rs7164594* as a genetic instrument were significant. Based on these results, the effect of HYKK on IA risk can be summarized in three ways. First, HYKK could negatively affect the IA risk mediated by cigarettes/day. Second, HYKK could also have a direct negative effect on IA risk. Third, HYKK could further reduce the risk of IA by its positive effect on the continuous abstinence of varenicline on smoking cessation.

### Limitations

This study has some limitations. First, not all genes that interacted with smoking cessation medications had eQTL data for analysis in the databases used in the current study. Therefore, the effects of these genes on IA were limited. Second, this study used only cis-eQTLs in the analyses, indicating that most variants influencing gene expression were near each other. Although various sensitivity analyses were performed to test the assumptions of the MR study, confounding biases and/or horizontal pleiotropy could not be completely excluded. Additionally, the current study was performed using a dataset with 7495 cases of IAs, and this could potentially explain the lack of association of some genetic instruments with the risk of IAs. Further analyses are still needed. Finally, the eQTLs and GWAS data used in this study were predominantly obtained from European populations; thus, these findings should be cautiously interpreted when generalizing them to other populations.

## CONCLUSIONS

This study investigated the association between IAs and smoking behavior and the pleiotropy between IAs and smoking cessation medications. The study observed that smoking initiation on IAs and cigarettes/day had causal effects on increasing IA risk and a causal effect of increased HYKK gene expression on lowering IA risk. This effect can be explained by the increased number of cigarettes consumed daily. HYKK could also further reduce the risk of IA due to the positive effect of continuous abstinence and varenicline administration on smoking cessation.

Additionally, the direct effect of HYKK on IA risk should not be ruled out, although it does not appear to be the main effect. Finally, this study found no direct evidence in the current data that smoking cessation medications adversely affected IAs. Follow-up studies are required to investigate the direction of the effect of varenicline on the HYKK gene, further analyze the effect of varenicline on IA risk, and whether and how variation in HYKK expression directly influences IAs. This study highlights the potential administration of smoking cessation medications for first-line and secondary prevention of IAs.

## Supplementary Material



## Data Availability

Data sharing is not applicable to this article as no new data were created.
