# Supplementary material for: Effect of smoking cessation medications on intracranial aneurysm risk: A Mendelian randomization study
Source: Tob Induc Dis. 2024 Apr 30;22:10.18332/tid/186171. doi: 10.18332/tid/186171 (PMC11059939; doi:10.18332/tid/186171)
Supplement: Supplementary file 1 [file TID-22-70-s1.pdf]

Supplementary Table S1 Mendelian randomization results of smoking behavior on IA risk

exposure	outcome	method	nsnp	beta	se	p-value	beta lower 95%CI	beta upper 95%CI
SmkAge	all IA	MR Egger	4	-3,120788299	3,326023117	0,447142836	-9,639793608	3,398217011
SmkAge	all IA	Weighted median	4	-0,854823528	0,758818053	0,259945584	-2,342106912	0,632459857
SmkAge	all IA	Inverse variance weighted	4	-0,613211069	0,845296439	0,468182707	-2,269992089	1,043569952
SmkAge	all IA	Simple mode	4	-1,461155495	1,424434703	0,380479401	-4,253047513	1,330736524
SmkAge	all IA	Weighted mode	4	-1,586716049	1,193852286	0,275862893	-3,926666529	0,753234432
CigDay	all IA	MR Egger	14	1,30707005	0,365240064	0,003791589	0,591199525	2,022940575
CigDay	all IA	Weighted median	14	1,259089	0,224847838	2,15E-08	0,818387238	1,699790762
CigDay	all IA	Inverse variance weighted	14	1,060856864	0,226823242	2,91E-06	0,61628331	1,505430417
CigDay	all IA	Simple mode	14	0,382474819	0,612932077	0,543417379	-0,818872052	1,58382169
CigDay	all IA	Weighted mode	14	1,283628204	0,237385272	0,000119625	0,81835307	1,748903337
SmkCes	all IA	MR Egger	3	17,27506515	24,00485351	0,602881839	-29,77444774	64,32457803
SmkCes	all IA	Weighted median	3	0,472360017	1,846465147	0,798090958	-3,146711672	4,091431705
SmkCes	all IA	Inverse variance weighted	3	1,917381599	3,347708033	0,566817655	-4,644126146	8,478889344
SmkCes	all IA	Simple mode	3	-1,992145745	2,588459556	0,521992104	-7,065526474	3,081234984
SmkCes	all IA	Weighted mode	3	-1,861402351	4,380498249	0,712238803	-10,44717892	6,724374218
SmkInt	all IA	MR Egger	65	1,136912356	1,45664876	0,43802025	-1,718119214	3,991943925
SmkInt	all IA	Weighted median	65	1,720127763	0,401601617	1,84E-05	0,932988593	2,507266932
SmkInt	all IA	Inverse variance weighted	65	1,533922859	0,287590363	9,62E-08	0,970245747	2,097599971
SmkInt	all IA	Simple mode	65	2,127197324	0,89202653	0,020067739	0,378825326	3,875569322
SmkInt	all IA	Weighted mode	65	1,789675241	0,693348239	0,012147262	0,430712693	3,148637789
SmkAge	only SAH	MR Egger	4	-1,962054203	2,826787424	0,559407116	-7,502557554	3,578449148
SmkAge	only SAH	Weighted median	4	-0,914789194	0,842341144	0,277475467	-2,565777836	0,736199449
SmkAge	only SAH	Inverse variance weighted	4	-0,33319956	0,700588493	0,634359709	-1,706353007	1,039953887
SmkAge	only SAH	Simple mode	4	-0,932521793	1,012852758	0,425113916	-2,9177132	1,052669613
SmkAge	only SAH	Weighted mode	4	-0,939484612	0,978907445	0,407992169	-2,858143204	0,97917398
CigDay	only SAH	MR Egger	14	1,319503045	0,363070494	0,003423701	0,607884876	2,031121214
CigDay	only SAH	Weighted median	14	1,220058155	0,270787731	6,62E-06	0,689314202	1,750802109
CigDay	only SAH	Inverse variance weighted	14	1,062000533	0,228430588	3,33E-06	0,61427658	1,509724486
CigDay	only SAH	Simple mode	14	0,894216301	0,681246356	0,212013454	-0,441026557	2,229459159
CigDay	only SAH	Weighted mode	14	1,277383157	0,279179958	0,000520403	0,73019044	1,824575875
SmkCes	only SAH	MR Egger	3	16,83083662	23,14137507	0,599682435	-28,52625852	62,18793175
SmkCes	only SAH	Weighted median	3	1,035907057	1,902249259	0,58604959	-2,692501491	4,764315604

SmkCes	only SAH	Inverse variance weighted	3	2,143775177	3,205732471	0,503666592	-4,139460466	8,42701082
SmkCes	only SAH	Simple mode	3	-1,468302068	3,538206195	0,718433409	-8,403186211	5,466582075
SmkCes	only SAH	Weighted mode	3	4,552800108	4,720712087	0,436585701	-4,699795582	13,8053958
SmkInt	only SAH	MR Egger	65	2,536072261	1,722320984	0,145871513	-0,839676868	5,911821389
SmkInt	only SAH	Weighted median	65	1,669100607	0,470009274	0,000383468	0,74788243	2,590318783
SmkInt	only SAH	Inverse variance weighted	65	1,383628499	0,341800917	5,16E-05	0,7136987	2,053558297
SmkInt	only SAH	Simple mode	65	2,03144682	1,008911324	0,048270807	0,053980625	4,008913014
SmkInt	only SAH	Weighted mode	65	1,923467826	0,897849449	0,035979182	0,163682905	3,683252747
SmkAge	only uIA	MR Egger	4	-7,467590083	7,768571346	0,437853415	-22,69398992	7,758809755
SmkAge	only uIA	Weighted median	4	-1,99278178	1,404710564	0,156003336	-4,746014485	0,760450925
SmkAge	only uIA	Inverse variance weighted	4	-1,001625718	1,85259847	0,588742018	-4,632718719	2,629467284
SmkAge	only uIA	Simple mode	4	-2,866502254	2,591696745	0,349438304	-7,946227874	2,213223366
SmkAge	only uIA	Weighted mode	4	-3,127138779	1,840177082	0,187811135	-6,733885859	0,4796083
CigDay	only uIA	MR Egger	14	1,198738008	0,720148932	0,121871214	-0,212753899	2,610229914
CigDay	only uIA	Weighted median	14	1,256767535	0,444527918	0,004695758	0,385492816	2,128042253
CigDay	only uIA	Inverse variance weighted	14	1,041012694	0,431103913	0,01574556	0,196049024	1,885976364
CigDay	only uIA	Simple mode	14	0,759930103	0,98712363	0,455155169	-1,174832212	2,694692417
CigDay	only uIA	Weighted mode	14	1,230308561	0,424177856	0,012397098	0,398919963	2,061697159
SmkCes	only uIA	MR Egger	3	18,798657	24,54172337	0,583870346	-29,3031208	66,9004348
SmkCes	only uIA	Weighted median	3	-0,727539864	2,591999701	0,778950704	-5,807859278	4,352779549
SmkCes	only uIA	Inverse variance weighted	3	0,612952577	3,60830553	0,865110257	-6,459326263	7,685231416
SmkCes	only uIA	Simple mode	3	-4,03447961	4,395400019	0,455575516	-12,64946365	4,580504427
SmkCes	only uIA	Weighted mode	3	-4,248884448	5,841297288	0,542613551	-15,69782713	7,200058238
SmkInt	only uIA	MR Egger	65	-2,612065452	2,388125834	0,278219447	-7,292792087	2,068661183
SmkInt	only uIA	Weighted median	65	1,588703044	0,67977474	0,01943387	0,256344553	2,921061534
SmkInt	only uIA	Inverse variance weighted	65	1,921447003	0,472852509	4,83E-05	0,994656086	2,84823792
SmkInt	only uIA	Simple mode	65	1,750129564	1,583861307	0,273306579	-1,354238598	4,854497726
SmkInt	only uIA	Weighted mode	65	1,633762937	1,236315291	0,191046894	-0,789415034	4,056940908

or	or lower 95%CI	or upper 95%CI
0,044122373	6,51E-05	29,91072198
0,425358248	0,096124898	1,882234918
0,54160893	0,103312997	2,839335234
0,231968082	0,01422083	3,783829242
0,204596394	0,019709263	2,123858395
3,6953307	1,806153642	7,560524564
3,522211291	2,266841012	5,472802156
2,888845275	1,852031806	4,506092713
1,465907961	0,440928719	4,873545447
3,609712768	2,266763561	5,748295274
31802807,98	1,17E-13	8,63E+27
1,603774666	0,042993271	59,82548289
6,803121826	0,009617931	4812,102336
0,136402426	0,000854045	21,78529028
0,155454476	2,90E-05	832,4508805
3,117128906	0,17940325	54,16007021
5,585242004	2,542095124	12,27134577
4,636328858	2,638592806	8,146594365
8,391315684	1,46056789	48,21013757
5,987507655	1,538353507	23,30429758
0,140569366	0,000551672	35,8179494
0,400601066	0,076859374	2,087984922
0,71662717	0,181526613	2,829086555
0,393559981	0,054057164	2,865290134
0,390829212	0,057375195	2,662256257
3,741561527	1,836542772	7,622628166
3,387384722	1,992348716	5,759220344
2,892151049	1,848319004	4,525483787
2,445418566	0,64337562	9,294837687
3,587240189	2,075475824	6,200164813
20395740,08	4,09E-13	1,02E+27
2,817660855	0,067711348	117,2508438

8,531585153	0,015931445	4568,822627
0,230316215	0,000224152	236,649957
94,89776063	0,009097137	989936,2225
12,62996621	0,431850045	369,3783248
5,307392218	2,112521865	13,33402159
3,989350751	2,041528313	7,79559084
7,625110542	1,055464153	55,08695925
6,844653431	1,177840769	39,77556374
0,000571303	1,39E-10	2342,115254
0,136315697	0,008686245	2,13924064
0,367281859	0,009728275	13,86638109
0,056897592	0,000353995	9,145147088
0,043843063	0,0011899	1,61544151
3,315929603	0,80835505	13,60217782
3,514044083	1,470338749	8,398408751
2,832083597	1,216586546	6,592788263
2,138126766	0,3088708	14,80096553
3,422285356	1,490214341	7,859296972
145932829,5	1,88E-13	1,13E+29
0,483096011	0,003003854	77,69411769
1,845873443	0,00156585	2175,97347
0,017694886	3,21E-06	97,56359549
0,014280155	1,52E-07	1339,508772
0,073382818	0,000680426	7,914220325
4,897393102	1,292197882	18,56098012
6,830835562	2,70379431	17,25734621
5,755348314	0,258143771	128,3162251
5,123116458	0,454110357	57,79723329

Supplementary Table S2 Cochran's Q test results of smoking behavior on IA risk

Exposure	Outcome	method	Q	df	pval
CigDay	all IA	MR Egger	16,1734028	12	0,18342237
CigDay	all IA	Inverse variance weighted	17,1828593	13	0,19107909
SmkInt	all IA	MR Egger	73,8782593	63	0,16428912
SmkInt	all IA	Inverse variance weighted	73,9689568	64	0,18475676
CigDay	only SAH	MR Egger	6,54565971	12	0,88612206
CigDay	only SAH	Inverse variance weighted	7,37825432	13	0,88162124
SmkInt	only SAH	MR Egger	75,275369	63	0,13825386
SmkInt	only SAH	Inverse variance weighted	75,8324566	64	0,14785495
CigDay	only uIA	MR Egger	18,4895963	12	0,10161317
CigDay	only uIA	Inverse variance weighted	18,609915	13	0,13570376
SmkInt	only uIA	MR Egger	48,9159697	63	0,90355231
SmkInt	only uIA	Inverse variance weighted	52,6667731	64	0,8432595

Supplementary Table S3 Results of MR-Egger regression and MR Pleiotropy RESidual Sum and Outlier tests

exposure	outcome	Egger intercept	se	pval	MR-PRESSO Global test RSSobs	MR-PRESSO Global test p-value
CigDay	all IA	-0,0111499	0,01288359	0,40377122	25,63326	0,2543333
SmkInt	all IA	0,004592066	0,016511949	0,781841992	76,08179	0,2103333
CigDay	only SAH	-0,01181121	0,012944271	0,379490144	13,63206	0,701
SmkInt	only SAH	-0,013325814	0,019515881	0,497224519	78,152	0,151
CigDay	only uIA	-0,006890344	0,02465741	0,784662817	20,91812	0,242
SmkInt	only uIA	0,052478961	0,027097117	0,057270005	54,132	0,8553333

Supplementary Table S4 Genes that interacted with smoking cessation medicat

Drug	Gene	Source
BUPROPION	GPRC6A	Drug Target (CMap, negative connectivity)
BUPROPION	ITGB4	Drug Target (CMap, negative connectivity)
BUPROPION	ITSN1	Drug Target (CMap, negative connectivity)
BUPROPION	ESYT1	Drug Target (CMap, negative connectivity)
BUPROPION	MAST4	Drug Target (CMap, negative connectivity)
BUPROPION	MAP3K5	Drug Target (CMap, negative connectivity)
BUPROPION	ATP1A1	Drug Target (CMap, negative connectivity)
BUPROPION	IL10	Drug Target (CMap, negative connectivity)
BUPROPION	CTSK	Drug Target (CMap, negative connectivity)
BUPROPION	LEF1	Drug Target (CMap, negative connectivity)
BUPROPION	ATG4A	Drug Target (CMap, negative connectivity)
BUPROPION	ZNF595	Drug Target (CMap, negative connectivity)
BUPROPION	RBBP7	Drug Target (CMap, negative connectivity)
BUPROPION	PRDM1	Drug Target (CMap, negative connectivity)
BUPROPION	CEBPB	Drug Target (CMap, negative connectivity)
BUPROPION	EED	Drug Target (CMap, negative connectivity)
BUPROPION	S100B	Drug Target (CMap, negative connectivity)
BUPROPION	ATG16L1	Drug Target (CMap, negative connectivity)
BUPROPION	FRAT2	Drug Target (CMap, negative connectivity)
BUPROPION	CASD1	Drug Target (CMap, negative connectivity)
BUPROPION	PRR7	Drug Target (CMap, positive connectivity)
BUPROPION	CDKN1C	Drug Target (CMap, positive connectivity)
BUPROPION	WARS2	Drug Target (CMap, positive connectivity)
BUPROPION	SUPT16H	Drug Target (CMap, positive connectivity)
BUPROPION	ZPBP2	Drug Target (CMap, positive connectivity)
BUPROPION	DUT	Drug Target (CMap, positive connectivity)
BUPROPION	LIPH	Drug Target (CMap, positive connectivity)
BUPROPION	FGFR2	Drug Target (CMap, positive connectivity)
BUPROPION	NR3C1	Drug Target (CMap, positive connectivity)
BUPROPION	HEXB	Drug Target (CMap, positive connectivity)
BUPROPION	ICAM1	Drug Target (CMap, positive connectivity)
BUPROPION	KARS	Drug Target (CMap, positive connectivity)
BUPROPION	KRT19	Drug Target (CMap, positive connectivity)
BUPROPION	NAB2	Drug Target (CMap, positive connectivity)
BUPROPION	PIK3R1	Drug Target (CMap, positive connectivity)
BUPROPION	ANKRD49	Drug Target (CMap, positive connectivity)

BUPROPION	RET	Drug Target (CMap, positive connectivity)
BUPROPION	SPP1	Drug Target (CMap, positive connectivity)
BUPROPION	USP32	Drug Target (CMap, positive connectivity)
BUPROPION	UMODL1	Drug Target (CMap, positive connectivity)
VARENICLINE	HMGB2	Drug Target (CMap, positive connectivity)
VARENICLINE	SLC25A1	Drug Target (CMap, positive connectivity)
VARENICLINE	GPR107	Drug Target (CMap, positive connectivity)
VARENICLINE	RPS13	Drug Target (CMap, positive connectivity)
VARENICLINE	P2RY8	Drug Target (CMap, positive connectivity)
VARENICLINE	MPI	Drug Target (CMap, positive connectivity)
VARENICLINE	SMU1	Drug Target (CMap, positive connectivity)
VARENICLINE	PIK3CG	Drug Target (CMap, positive connectivity)
VARENICLINE	PAICS	Drug Target (CMap, positive connectivity)
VARENICLINE	FIS1	Drug Target (CMap, positive connectivity)
VARENICLINE	PGK1	Drug Target (CMap, positive connectivity)
VARENICLINE	PHACTR1	Drug Target (CMap, positive connectivity)
VARENICLINE	TLR5	Drug Target (CMap, positive connectivity)
VARENICLINE	CYB5B	Drug Target (CMap, positive connectivity)
VARENICLINE	PFKL	Drug Target (CMap, positive connectivity)
VARENICLINE	TFF2	Drug Target (CMap, positive connectivity)
VARENICLINE	FST	Drug Target (CMap, positive connectivity)
VARENICLINE	GPR65	Drug Target (CMap, positive connectivity)
VARENICLINE	PTP4A1	Drug Target (CMap, positive connectivity)
VARENICLINE	SMAD2	Drug Target (CMap, positive connectivity)
VARENICLINE	HEATR1	Drug Target (CMap, negative connectivity)
VARENICLINE	NAT10	Drug Target (CMap, negative connectivity)
VARENICLINE	CHRNA4	Drug Target (Drug Gene Interaction database)
VARENICLINE	CHRNB2	Drug Target (Drug Gene Interaction database)
VARENICLINE	CHRNA3	Drug Target (Drug Gene Interaction database)
VARENICLINE	CHRNB4	Drug Target (Drug Gene Interaction database)
VARENICLINE	HYKK	Drug Target (Drug Gene Interaction database)
VARENICLINE	CHRNA7	Drug Target (Drug Gene Interaction database)
VARENICLINE	CYP2A6	Drug Target (Drug Gene Interaction database)
BUPROPION	DBH	Drug Target (Drug Gene Interaction database)
BUPROPION	PRL	Drug Target (Drug Gene Interaction database)
BUPROPION	SLC18A2	Drug Target (Drug Gene Interaction database)
BUPROPION	SLC6A3	Drug Target (Drug Gene Interaction database)
BUPROPION	JUN	Drug Target (Drug Gene Interaction database)

BUPROPION	CYP2A6	Drug Target (Drug Gene Interaction database)
BUPROPION	NAT1	Drug Target (Drug Gene Interaction database)
BUPROPION	SLC6A2	Drug Target (Drug Gene Interaction database)
BUPROPION	EPB41	Drug Target (Drug Gene Interaction database)
BUPROPION	HTR2A	Drug Target (Drug Gene Interaction database)
BUPROPION	DRD1	Drug Target (Drug Gene Interaction database)
BUPROPION	ANKK1	Drug Target (Drug Gene Interaction database)
BUPROPION	GALR1	Drug Target (Drug Gene Interaction database)
BUPROPION	ACE	Drug Target (Drug Gene Interaction database)
BUPROPION	FKBP5	Drug Target (Drug Gene Interaction database)
BUPROPION	SLC6A4	Drug Target (Drug Gene Interaction database)
BUPROPION	DRD2	Drug Target (Drug Gene Interaction database)
BUPROPION	COMT	Drug Target (Drug Gene Interaction database)
BUPROPION	SACM1L	Drug Target (Drug Gene Interaction database)

Supplementary Table S5 Summary-data-based Mendelian randomization results of medication targets on IA risk

Gene	Tissue	Top SNP	Top SNP CHR	SMR Beta	SMR OR	SMR p-value
ACE	Nerve Tibial	rs7213524	17	-0,0831178	0,920242736	0,4728549
ACE	Thyroid	rs12449782	17	0,0463857	1,047478345	0,7480564
ACE	Colon Transverse	rs4311	17	0,012409	1,012486311	0,8356426
ACE	Blood	rs4311	17	-0,0766177	0,926243889	0,8356599
ACE	Skin Sun Exposed Lower leg	rs4311	17	-0,0291277	0,971292423	0,8356599
ACE	Skin Not Sun Exposed Suprapubic	rs4311	17	-0,0275132	0,972861841	0,835681
ACE	Adipose Subcutaneous	rs4311	17	-0,0329889	0,967549299	0,8356838
ACE	Spleen	rs2440144	17	0,00527459	1,005288525	0,9206863
ACE	Lung	rs4325	17	-0,00529898	0,994715035	0,9376222
ACE	Cells Cultured fibroblasts	rs4344	17	-0,0069093	0,993114514	0,9442996
ANKK1	Whole blood	rs11214603	11	-0,16921	0,844331575	0,004852966
ANKK1	Lung	rs6277	11	-0,200659	0,818191387	0,00565371
ANKK1	Colon Sigmoid	rs10891549	11	-0,150474	0,860300098	0,006075508
ANKK1	Adipose Visceral Omentum	rs34601878	11	-0,172313	0,841715675	0,006748397
ANKK1	Nerve Tibial	rs2186799	11	-0,0836955	0,919711266	0,01365374
ANKK1	Muscle Skeletal	rs2186800	11	-0,142881	0,866857219	0,01496616
ANKK1	Pancreas	rs11214589	11	-0,126104	0,881523164	0,01764031
ANKK1	Liver	rs2186800	11	-0,125839	0,881756798	0,01998491
ANKK1	Small Intestine Terminal Ileum	rs11214589	11	-0,185943	0,830320922	0,0209619
ANKK1	Esophagus Mucosa	rs1078	11	0,201723	1,223509049	0,02107885
ANKK1	Heart Left Ventricle	rs4938012	11	-0,124698	0,882763457	0,02332707
ANKK1	Heart Atrial Appendage	rs7123797	11	-0,132614	0,875803087	0,02743452
ANKK1	Stomach	rs7942392	11	-0,0890055	0,914840542	0,1471669
ANKK1	Artery Aorta	rs2282511	11	-0,0813504	0,921870611	0,1515279
ANKK1	Blood	rs6589371	11	-0,120405	0,886561307	0,1543394
ANKK1	Adipose Subcutaneous	rs6589371	11	-0,0763045	0,926534034	0,1587454
ANKK1	Esophagus Muscularis	rs6589371	11	-0,0827074	0,920620481	0,1598929
ANKK1	Brain Frontal Cortex BA9	rs6589371	11	-0,0429618	0,957947983	0,1601646
ANKK1	Brain Cortex	rs6589371	11	-0,0491945	0,951995948	0,1602232
ANKK1	Artery Coronary	rs73572791	11	-0,101719	0,903283339	0,1607228
ANKK1	Brain Cerebellum	rs6589371	11	-0,0471312	0,95396223	0,1609303
ANKK1	Colon Transverse	rs6589371	11	-0,0920739	0,912037747	0,1626107

ANKK1	Brain Anterior cingulate cortex BA24	rs6589371	11	-0,0489358	0,952242262	0,1628185
ANKK1	Ovary	rs6589371	11	-0,0679899	0,93426991	0,1630167
ANKK1	Spleen	rs6589371	11	-0,0785586	0,924447886	0,1634021
ANKK1	Artery Tibial	rs6589371	11	-0,110682	0,895223385	0,1636055
ANKK1	Brain Cerebellar Hemisphere	rs6589371	11	-0,0545146	0,946944683	0,163656
ANKK1	Esophagus Gastroesophageal Junction	rs6589371	11	-0,0823051	0,920990921	0,1637956
ANKK1	Testis	rs7130431	11	-0,058961	0,942743535	0,176232
ANKK1	Thyroid	rs2298489	11	-0,135177	0,873561278	0,1977341
ANKK1	Cells Cultured fibroblasts	rs75343922	11	0,00338591	1,003391649	0,9506788
ANKRD49						
ATG16L1	Blood	rs10192702	2	0,126469	1,134814271	0,3113544
ATG16L1	Adipose Subcutaneous	rs7559194	2	-0,100108	0,904739701	0,3123163
ATG16L1	Cells Cultured fibroblasts	rs2289477	2	0,177323	1,194016698	0,3299825
ATG16L1	Skin Not Sun Exposed Suprapubic	rs6745825	2	0,0534607	1,054915533	0,3462154
ATG16L1	Testis	rs6745825	2	0,0733556	1,076113135	0,3463568
ATG16L1	Thyroid	rs56227616	2	0,148348	1,159916477	0,3664998
ATG16L1	Skin Sun Exposed Lower leg	rs6431659	2	-0,0504838	0,950769331	0,4166514
ATG16L1	Nerve Tibial	rs11685932	2	0,0797998	1,083070215	0,4223416
ATG16L1	Minor Salivary Gland	rs6737398	2	-0,0544811	0,946976407	0,4614773
ATG16L1	Esophagus Mucosa	rs3816234	2	0,030528	1,030998758	0,5057285
ATG16L1	Muscle Skeletal	rs7594365	2	-0,0738212	0,928837755	0,6377532
ATG4A						
ATP1A1	Esophagus Mucosa	rs10924046	1	0,0502706	1,051555609	0,6941962
CASD1	Testis	rs10250095	7	0,155651	1,168418354	0,07635438
CASD1	Cells Cultured fibroblasts	rs1056413	7	-0,149092	0,861489854	0,2709465
CASD1	Nerve Tibial	rs3763464	7	0,0949842	1,099641481	0,4314388
CASD1	Blood	rs2301645	7	0,157224	1,170257722	0,5865914
CASD1	Thyroid	rs2301645	7	0,0395856	1,040379552	0,5866108
CASD1	Esophagus Mucosa	rs2301645	7	0,039891	1,040697332	0,5866259
CASD1	Adipose Subcutaneous	rs2301645	7	0,0611462	1,063054321	0,5870059
CASD1	Muscle Skeletal	rs2301645	7	0,0662356	1,068478421	0,587139
CASD1	Artery Tibial	rs2301645	7	0,0650816	1,067246108	0,5871688
CASD1	Esophagus Muscularis	rs2301645	7	0,0478564	1,049020005	0,5872513
CASD1	Pancreas	rs2301645	7	0,037316	1,038020984	0,5874339

CASD1	Artery Aorta	rs2301645	7	0,0444422	1,045444548	0,5874356
CASD1	Lung	rs2301645	7	0,0777102	1,080809395	0,5877659
CDKN1C	Nerve Tibial	rs17743926	11	0,106421	1,112290052	0,3283234
CDKN1C	Blood	rs234854	11	-0,00431688	0,995692424	0,9852252
CEBPB	Blood	rs1889236	20	0,288362	1,334240212	0,100263
CHRNA3	Colon Sigmoid	rs17486278	15	-0,417033	0,658999173	0,000134841
CHRNA3	Testis	rs1809415	15	0,114565	1,121385529	0,0420711
CHRNA3	Nerve Tibial	rs4886572	15	-0,033988	0,966583104	0,2692013
CHRNA3	Muscle Skeletal	rs28392948	15	-0,0294269	0,971001855	0,2785703
CHRNA3	Brain Nucleus accumbens basal ganglia	rs950776	15	-0,0316234	0,96887139	0,2980649
CHRNA3	Brain Caudate basal ganglia	rs8023462	15	-0,0262385	0,974102738	0,3167217
CHRNA3	Brain Putamen basal ganglia	rs8023462	15	-0,0320717	0,968437143	0,3179268
CHRNA4	Spleen	rs58626340	20	0,050604	1,051906256	0,3249479
CHRNA4	Nerve Tibial	rs12624510	20	0,00909544	1,009136929	0,8234294
CHRNA4	Brain Putamen basal ganglia	rs12624510	20	0,0131542	1,013241097	0,8235199
CHRNA4	Brain Caudate basal ganglia	rs12624510	20	0,0157437	1,015868285	0,8235387
CHRNA4	Brain Nucleus accumbens basal ganglia	rs73157403	20	-0,00514767	0,994865557	0,9417159
CHRNA4	Brain Frontal Cortex BA9	rs73157403	20	-0,00681863	0,993204564	0,9417168
CHRNA4	Liver	rs3810471	20	0,000760252	1,000760541	0,9792781
CHRNA4	Testis	rs3787142	20	-0,00195481	0,998047099	0,9891448
CHRNA7	Skin Sun Exposed Lower leg	rs1425292	15	0,159855	1,173340724	0,03502589
CHRNA7	Artery Tibial	rs11639169	15	0,130642	1,139559746	0,06580833
CHRNA7	Lung	rs12440544	15	0,0659175	1,068138592	0,1984544
CHRNA7	Nerve Tibial	rs72713546	15	0,051063	1,052389192	0,2713768
CHRNA7	Pancreas	rs60109258	15	-0,0406295	0,960184813	0,4193846
CHRNA7	Adipose Subcutaneous	rs16956707	15	0,0385151	1,039266421	0,4948704
CHRNA7	Thyroid	rs34959140	15	0,0307593	1,031237255	0,5377552
CHRNA7	Artery Aorta	rs28522500	15	0,0230223	1,023289359	0,6554459
CHRNB2	Blood	rs11264222	1	-0,126776	0,880930979	0,4171514
CHRNB2	Whole blood	rs11264222	1	-0,0773131	0,925600003	0,4197647
CHRNB2	Skin Sun Exposed Lower leg	rs4845652	1	-0,0210509	0,979169124	0,7240819
CHRNB2	Skin Not Sun Exposed Suprapubic	rs4845652	1	-0,0322238	0,968289855	0,7243944
CHRNB2	Esophagus Mucosa	rs4845378	1	-0,00791711	0,992114148	0,8926722
CHRNB4	Esophagus Mucosa	rs4886580	15	0,170865	1,186330584	0,011763

CHRNB4	Testis	rs12912524	15	0,172035	1,187719403	0,03357038
COMT	Esophagus Mucosa	rs6269	22	0,0890194	1,093101862	0,1189013
COMT	Cells Cultured fibroblasts	rs45616631	22	-0,117203	0,889404626	0,169559
COMT	Esophagus Muscularis	rs45616631	22	-0,084457	0,919011172	0,1699839
COMT	Esophagus Gastroesophageal Junction	rs45616631	22	-0,0937767	0,910486051	0,1708661
COMT	Adipose Visceral Omentum	rs45616631	22	-0,0941894	0,910110371	0,1708991
COMT	Breast Mammary Tissue	rs45616631	22	-0,112093	0,893961115	0,1720169
COMT	Skin Not Sun Exposed Suprapubic	rs165722	22	0,160699	1,174331442	0,1724706
COMT	Heart Atrial Appendage	rs45616631	22	-0,101401	0,903570628	0,1729925
COMT	Brain Cerebellum	rs45616631	22	-0,101748	0,903257144	0,1754376
COMT	Brain Cerebellar Hemisphere	rs45616631	22	-0,104632	0,900655903	0,1778803
COMT	Pituitary	rs45616631	22	-0,0967203	0,907809885	0,1782762
COMT	Lung	rs45616631	22	-0,177592	0,837283966	0,178988
COMT	Nerve Tibial	rs45454096	22	-0,0924522	0,911692789	0,2411545
COMT	Heart Left Ventricle	rs45454096	22	-0,113625	0,892592615	0,2457466
COMT	Artery Tibial	rs73880021	22	-0,0981649	0,90649941	0,2630384
COMT	Adipose Subcutaneous	rs73880021	22	-0,0826314	0,920690451	0,2633287
COMT	Artery Aorta	rs73880021	22	-0,0806992	0,922471129	0,2640018
COMT	Colon Sigmoid	rs73880021	22	-0,0915896	0,912479554	0,2662565
COMT	Thyroid	rs6518591	22	-0,149691	0,860973976	0,3221623
COMT	Blood	rs9618717	22	-0,0822992	0,920996355	0,3280404
COMT	Cells EBV transformed lymphocytes	rs9618717	22	-0,0404458	0,960361215	0,333378
COMT	Pancreas	rs6518589	22	-0,0420776	0,958795375	0,5101149
COMT	Muscle Skeletal	rs6518589	22	-0,103295	0,901860885	0,5117297
COMT	Whole blood	rs174680	22	0,0342637	1,034857463	0,8507592
CTSK	Heart Left Ventricle	rs2134688	1	-0,197886	0,820463381	0,1963465
CTSK	Cells Cultured fibroblasts	rs3768017	1	-0,0961446	0,908332662	0,4477276
CTSK	Heart Atrial Appendage	rs3768017	1	-0,0772052	0,925699881	0,448499
CTSK	Testis	rs72704656	1	-0,0562139	0,945336907	0,5925897
CTSK	Brain Cerebellar Hemisphere	rs12126004	1	-0,0189864	0,981192706	0,6222927
CTSK	Artery Coronary	rs7535032	1	0,0103797	1,010433756	0,8912878
CTSK	Pancreas	rs12067685	1	-0,00843817	0,991597331	0,9251057
CTSK	Brain Cerebellum	rs11204737	1	-0,00152215	0,998479008	0,9643922
CTSK	Pituitary	rs11204737	1	-0,00294577	0,997058565	0,9643927

CTSK	Blood	rs2089081	1	0,00079709	1,000797408	0,9821916
CTSK	Whole blood	rs2039590	1	0,00107903	1,001079612	0,9857528
CTSK	Artery Aorta	rs12042263	1	0,000468399	1,000468509	0,9959703
CTSK	Liver	rs7534124	1	0	1	1
CYB5B	Esophagus Mucosa	rs246139	16	-0,258372	0,77230788	0,09426818
CYB5B	Whole blood	rs246147	16	0,341883	1,407595599	0,09876287
CYB5B	Skin Sun Exposed Lower leg	rs9929584	16	-0,114587	0,891734354	0,5042875
CYB5B	Skin Not Sun Exposed Suprapubic	rs62057899	16	-0,0793842	0,923684976	0,6021572
CYB5B	Blood	rs72797202	16	-0,0500149	0,951215251	0,7985059
CYP2A6	Ovary	rs3797218	19	0,0716027	1,074228468	0,1180923
CYP2A6	Ovary	rs3797218	19	0,0716027	1,074228468	0,1180923
DBH	Pancreas	rs2905214	9	-0,0271052	0,973258849	0,5515564
DBH	Thyroid	rs2905214	9	-0,0611853	0,940648921	0,552668
DBH	Stomach	rs491072	9	-0,0297588	0,970679633	0,5614521
DBH	Esophagus Mucosa	rs2519071	9	0,0325804	1,033116952	0,6694952
DBH	Blood	rs2519072	9	0,060744	1,062626847	0,683823
DBH	Brain Cortex	rs2519152	9	-0,022177	0,978067102	0,7338887
DBH	Nerve Tibial	rs9409872	9	0,010833	1,010891889	0,8532327
DBH	Adipose Visceral Omentum	rs2849750	9	0,0095581	1,009603925	0,878412
DBH	Colon Sigmoid	rs73548055	9	0,00321851	1,003223695	0,9601239
DBH	Muscle Skeletal	rs10993778	9	0,000685493	1,000685728	0,9938149
DBH	Esophagus Muscularis	rs10993775	9	0	1	1
DBH	Esophagus Gastroesophageal Junction	rs10993776	9	0	1	1
DRD1	Adipose Visceral Omentum	rs6556205	5	0,060532	1,062401594	0,3065853
DRD2	Nerve Tibial	rs3802856	11	-0,129825	0,878249111	0,02816802
DRD2	Kidney Cortex	rs2511052	11	-0,0482538	0,952891912	0,02944601
DRD2	Minor Salivary Gland	rs2465651	11	0,0986862	1,103719898	0,0319362
DRD2	Skin Not Sun Exposed Suprapubic	rs2465651	11	0,235429	1,265451531	0,03636979
DRD2	Skin Sun Exposed Lower leg	rs7110738	11	0,139938	1,150202484	0,04116093
DRD2	Muscle Skeletal	rs7114297	11	-0,0230903	0,977174241	0,6473981
DRD2	Esophagus Muscularis	rs6589381	11	-0,0102596	0,98979285	0,8621287
DRD2	Esophagus Gastroesophageal Junction	rs6589381	11	-0,0108529	0,98920578	0,8621484
DRD2	Stomach	rs7107293	11	-0,0113515	0,988712685	0,8627939
DRD2	Adipose Subcutaneous	rs4936272	11	-0,006115	0,993903659	0,9368026

DUT	Whole blood	rs76749119	15	-0,0382661	0,962456797	0,2985305
DUT	Blood	rs964611	15	-0,0883993	0,915395286	0,3429751
DUT	Skin Sun Exposed Lower leg	rs76539098	15	0,0868218	1,090702299	0,4864172
EED	Cells Cultured fibroblasts	rs291203	11	-0,0990501	0,905697331	0,4674494
EED	Whole blood	rs11234594	11	-0,0977112	0,906910782	0,4737241
EED	Spleen	rs10898463	11	-0,0396576	0,96111847	0,4996287
EED	Brain Cerebellum	rs1874694	11	0,0227462	1,023006867	0,5514314
EED	Blood	rs7942971	11	-0,0767839	0,92608996	0,6105382
EED	Brain Cerebellar Hemisphere	rs7942971	11	0,0280562	1,028453482	0,611726
EPB41	Blood	rs12354050	1	-0,256213	0,773977094	0,2990874
EPB41	Whole blood	rs157204	1	-0,257306	0,773131599	0,3234036
EPB41	Skin Sun Exposed Lower leg	rs157220	1	-0,173748	0,840508679	0,421385
EPB41	Testis	rs457618	1	-0,109188	0,896561848	0,4359171
EPB41	Brain Cerebellar Hemisphere	rs150085	1	-0,0196938	0,980498856	0,7018459
EPB41	Brain Cerebellum	rs150085	1	-0,0227601	0,977496957	0,7018889
ESYT1	Blood	rs2640569	12	0,215863	1,24093236	0,3192405
FGFR2	Colon Sigmoid	rs10788178	10	0,173823	1,189844945	0,1475228
FGFR2	Heart Atrial Appendage	rs1693679	10	-0,100902	0,904021623	0,1751914
FGFR2	Blood	rs1896422	10	0,051089	1,052416554	0,3555031
FGFR2	Artery Aorta	rs7079213	10	-0,0370433	0,963634409	0,5311079
FGFR2	Artery Tibial	rs10788185	10	-0,0328675	0,967666767	0,6012608
FGFR2	Adipose Visceral Omentum	rs73361168	10	0,0122708	1,012346395	0,872757
FIS1	Skin Not Sun Exposed Suprapubic	rs76238898	7	-0,0109358	0,989123778	0,9029415
FIS1	Esophagus Muscularis	rs76238898	7	-0,0120232	0,98804879	0,902944
FIS1	Thyroid	rs74662330	7	-0,0223465	0,977901334	0,9059757
FIS1	Cells Cultured fibroblasts	rs6964812	7	-0,00830206	0,991732307	0,9109933
FIS1	Adipose Subcutaneous	rs6964812	7	-0,0106549	0,989401662	0,9109934
FIS1	Skin Sun Exposed Lower leg	rs6964812	7	-0,0104287	0,98962549	0,9109934
FIS1	Artery Tibial	rs6964812	7	-0,0133966	0,986692735	0,9109939
FIS1	Nerve Tibial	rs6964812	7	-0,0114635	0,988601956	0,9109939
FIS1	Lung	rs6964812	7	-0,0113453	0,988718815	0,9109952
FIS1	Artery Aorta	rs6964812	7	-0,00982103	0,990227039	0,9109959
FIS1	Colon Transverse	rs6964812	7	-0,0107138	0,989343388	0,9109971
FIS1	Stomach	rs6964812	7	-0,0112327	0,988830151	0,9109986

FIS1	Ovary	rs6964812	7	-0,00851743	0,991518741	0,911
FIS1	Cells EBV transformed lymphocytes	rs6964812	7	-0,00675555	0,993267217	0,9110002
FIS1	Pancreas	rs6964812	7	-0,0116836	0,988384388	0,911004
FIS1	Uterus	rs9656066	7	-0,00539415	0,994620372	0,9249282
FIS1	Breast Mammary Tissue	rs6957526	7	-0,0114983	0,988567553	0,9333183
FIS1	Prostate	rs6957526	7	-0,00760023	0,992428579	0,9333209
FIS1	Blood	rs114756165	7	-0,00415592	0,995852704	0,9368193
FIS1	Testis	rs73713820	7	-0,00843744	0,991598055	0,9465263
FIS1	Artery Coronary	rs6979122	7	-0,00562278	0,994392998	0,9480382
FIS1	Adipose Visceral Omentum	rs73713821	7	-0,00690611	0,993117682	0,9481423
FIS1	Small Intestine Terminal Ileum	rs73713821	7	-0,00672936	0,993293231	0,9481439
FIS1	Esophagus Gastroesophageal Junction	rs113045567	7	-0,00277155	0,997232287	0,9788773
FIS1	Colon Sigmoid	rs113045567	7	-0,0027658	0,997238021	0,9788773
FIS1	Esophagus Mucosa	rs76485948	7	-0,00173895	0,998262561	0,9853757
FIS1	Whole blood	rs10241107	7	-0,00229765	0,997704988	0,9902302
FKBP5						
FRAT2	Testis	rs1890966	10	0,0922014	1,096585652	0,237577
FRAT2	Brain Frontal Cortex BA9	rs35717991	10	-0,0699827	0,93240995	0,2787848
FRAT2	Esophagus Mucosa	rs12769073	10	0,104631	1,110300834	0,3528666
FRAT2	Brain Cortex	rs29001262	10	-0,038042	0,962672508	0,5690365
FRAT2	Whole blood	rs10882904	10	-0,0826823	0,920643589	0,6335127
FRAT2	Blood	rs11189165	10	-0,0167399	0,983399434	0,6812873
FRAT2	Muscle Skeletal	rs7903562	10	-0,0178006	0,982356895	0,8647227
FRAT2	Lung	rs10882900	10	-0,0042968	0,995712418	0,9728437
FST	Blood	rs77333810	5	0,097194	1,102074155	0,2927521
GALR1	Nerve Tibial	rs55944549	18	0,137914	1,147876829	0,1022745
GALR1	Breast Mammary Tissue	rs2850892	18	-0,0810292	0,922166764	0,1338807
GALR1	Adipose Visceral Omentum	rs2850892	18	-0,0727612	0,929822845	0,1340183
GALR1	Esophagus Gastroesophageal Junction	rs2850892	18	-0,0848652	0,918636108	0,1367748
GALR1	Adipose Subcutaneous	rs11662010	18	-0,0682115	0,934062898	0,1374073
GALR1	Esophagus Muscularis	rs72978691	18	-0,0887426	0,915081085	0,1377292
GALR1	Skin Sun Exposed Lower leg	rs72978691	18	-0,102836	0,902274934	0,1407092
GALR1	Thyroid	rs2850912	18	-0,0787766	0,924246378	0,2422667
GALR1	Pituitary	rs2850909	18	-0,001628	0,998373324	0,9775276

GPR107	Blood	rs61456260	9	-0,0238731	0,976409608	0,9524739
GPR65	Blood	rs6574976	14	-0,166201	0,846875995	0,04308903
GPR65	Whole blood	rs77194943	14	-0,159673	0,852422486	0,1335297
GPR65	Cells Cultured fibroblasts	rs116899835	14	-0,0466648	0,954407261	0,4227271
GPRC6A						
HEATR1	Testis	rs10802552	1	0,266019	1,304759849	0,04621676
HEATR1	Colon Sigmoid	rs1773451	1	0,249286	1,28310895	0,06739561
HEATR1	Esophagus Muscularis	rs1126627	1	0,219789	1,245813836	0,06961566
HEATR1	Blood	rs4233465	1	0,31351	1,368219145	0,143571
HEATR1	Muscle Skeletal	rs4233465	1	0,317087	1,373122028	0,1488923
HEATR1	Lung	rs4233465	1	0,350174	1,419314488	0,1547348
HEATR1	Esophagus Gastroesophageal Junction	rs2794763	1	0,16758	1,182439882	0,2538373
HEATR1	Brain Cerebellum	rs2853612	1	0,0672122	1,069522407	0,2745494
HEATR1	Artery Tibial	rs2853612	1	0,170071	1,185389011	0,2753318
HEATR1	Brain Cerebellar Hemisphere	rs2853612	1	0,09847	1,103481299	0,2766401
HEXB	Whole blood	rs10474420	5	-0,344313	0,708707068	0,01746482
HEXB	Blood	rs10075142	5	-0,202651	0,816563172	0,01774599
HMGB2	Blood	rs13128793	4	0,0453803	1,04642574	0,8545527
HTR2A	Minor Salivary Gland	rs13378925	13	-0,026005	0,974330218	0,5019048
HTR2A	Artery Aorta	rs9591060	13	-0,0224534	0,977796801	0,6358478
HTR2A	Testis	rs9534515	13	-0,0136454	0,986447276	0,7063822
HTR2A	Colon Sigmoid	rs11148042	13	-0,0270216	0,973340217	0,7154554
HTR2A	Esophagus Mucosa	rs35186029	13	-0,0258393	0,974491678	0,7654659
HTR2A	Artery Tibial	rs35148622	13	-0,0182359	0,981929368	0,7925509
HTR2A	Lung	rs12872365	13	-0,0112182	0,988844489	0,81557
HTR2A	Esophagus Muscularis	rs12872365	13	-0,0144385	0,985665235	0,8156011
HTR2A	Adipose Subcutaneous	rs2104936	13	-0,0240604	0,976226744	0,8280162
HYKK	Blood	rs931794	15	-2,078	0,125180323	8,73913E-05
HYKK	Brain Caudate basal ganglia	rs4887062	15	0,0601549	1,062001038	0,2515964
HYKK	Brain Cortex	rs2869544	15	0,0692451	1,07169885	0,2728862
HYKK	Heart Left Ventricle	rs7173512	15	0,0838138	1,087426396	0,3650745
ICAM1	Blood	rs281437	19	-0,206958	0,813053798	0,09755327
ICAM1	Whole blood	rs281437	19	-0,254534	0,775277693	0,1076722
IL10	Whole blood	rs1518110	1	0,157079	1,170088047	0,1720145

IL10	Blood	rs1518111	1	0,192109	1,211802596	0,1749097
ITGB4	Nerve Tibial	rs9910627	17	0,297566	1,346577247	0,04094785
ITGB4	Pancreas	rs4789223	17	-0,130156	0,877958459	0,1009334
ITGB4	Stomach	rs2291031	17	-0,093857	0,910412942	0,4983429
ITSN1	Brain Caudate basal ganglia	rs2244966	21	0,0699391	1,072442867	0,419055
ITSN1	Whole blood	rs2300375	21	-0,0525508	0,94880612	0,505004
ITSN1	Blood	rs4817592	21	-0,0231622	0,977103985	0,5182815
ITSN1	Skin Not Sun Exposed Suprapubic	rs9981693	21	0,0591803	1,060966516	0,528894
ITSN1	Skin Sun Exposed Lower leg	rs9974830	21	0,0703762	1,072911735	0,5381375
ITSN1	Artery Aorta	rs9978415	21	-0,0295955	0,970838158	0,7475826
ITSN1	Artery Tibial	rs2834282	21	-0,0178964	0,98226279	0,8839292
ITSN1	Cells Cultured fibroblasts	rs2246647	21	-0,0178312	0,982326835	0,9192058
JUN	Blood	rs2716140	1	0,124001	1,132017003	0,2169653
KARS						
KRT19	Brain Cerebellar Hemisphere	rs16966660	17	-0,0143433	0,985759075	0,6822692
KRT19	Thyroid	rs1823996	17	-0,0345971	0,965994537	0,6994966
KRT19	Cells Cultured fibroblasts	rs9905005	17	-0,00581379	0,994203077	0,943257
KRT19	Testis	rs9303313	17	0,00185411	1,00185583	0,9697082
KRT19	Esophagus Mucosa	rs9303313	17	0,00378159	1,003788749	0,9697082
KRT19	Brain Cerebellum	rs73307180	17	-0,000466803	0,999533306	0,9922537
LEF1	Heart Left Ventricle	rs140355156	4	-0,0724701	0,930093556	0,1876417
LIPH	Cells Cultured fibroblasts	rs79952553	3	0,147525	1,158962259	0,08641804
LIPH	Testis	rs55797844	3	-0,148737	0,861795737	0,200535
LIPH	Blood	rs79465457	3	0,152314	1,16452584	0,6739923
MAP3K5	Lung	rs6924387	6	0,117983	1,125224982	0,2117779
MAP3K5	Adipose Visceral Omentum	rs6924387	6	0,13633	1,146060031	0,214457
MAP3K5	Brain Cerebellum	rs9483947	6	-0,0394352	0,961332246	0,405737
MAP3K5	Blood	rs6899530	6	0,035752	1,036398788	0,4572647
MAP3K5	Artery Tibial	rs9402828	6	0,00895783	1,008998071	0,9298252
MAP3K5	Brain Cerebellar Hemisphere	rs59122276	6	0,000382397	1,00038247	0,993617
MAST4	Thyroid	rs17283091	5	0,139231	1,149389578	0,2104774
MAST4	Nerve Tibial	rs455788	5	-0,121862	0,885270527	0,3740496
MAST4	Colon Sigmoid	rs469394	5	-0,0488199	0,952352633	0,4849166
MAST4	Colon Transverse	rs469394	5	-0,069535	0,932827484	0,485278

MAST4	Cells Cultured fibroblasts	rs2662226	5	-0,0501316	0,951104251	0,6364256
MAST4	Pancreas	rs58331610	5	0,0382232	1,038963104	0,6755068
MAST4	Esophagus Muscularis	rs1428408	5	-0,0350423	0,965564572	0,7079309
MAST4	Muscle Skeletal	rs584354	5	-0,0185072	0,981663007	0,7142429
MAST4	Artery Tibial	rs250277	5	-0,0231474	0,977118446	0,7174367
MAST4	Heart Left Ventricle	rs2096416	5	0,00344896	1,003454915	0,9257301
MAST4	Heart Atrial Appendage	rs2096416	5	0,00710032	1,007125587	0,925737
MAST4	Whole blood	rs10940094	5	0,00537699	1,005391472	0,9356693
MAST4	Blood	rs10471698	5	0,00264831	1,00265182	0,9642325
MAST4	Esophagus Gastroesophageal Junction	rs7735834	5	-0,00196725	0,998034684	0,9799836
MPI	Artery Tibial	rs12911254	15	0,16052	1,174121255	0,3041759
MPI	Esophagus Muscularis	rs12901190	15	0,0994823	1,104598919	0,3284959
MPI	Nerve Tibial	rs4886608	15	0,0835105	1,08709663	0,3326686
MPI	Breast Mammary Tissue	rs4886608	15	0,0841835	1,087828492	0,3358238
MPI	Esophagus Gastroesophageal Junction	rs4886632	15	0,0976358	1,102561159	0,3434096
MPI	Brain Cerebellum	rs12912839	15	0,0760952	1,079065296	0,3541618
MPI	Brain Nucleus accumbens basal ganglia	rs11072511	15	0,0532809	1,054725876	0,3583937
MPI	Thyroid	rs11072511	15	0,0970878	1,101957121	0,3584038
MPI	Brain Hippocampus	rs11072511	15	0,0637224	1,065796493	0,3609357
MPI	Blood	rs1130741	15	0,0722162	1,07488771	0,3651804
MPI	Esophagus Mucosa	rs12903205	15	0,0880441	1,09203628	0,3653341
MPI	Cells Cultured fibroblasts	rs1130741	15	0,0579785	1,059692212	0,3662516
MPI	Whole blood	rs1130741	15	0,105074	1,110792806	0,3668479
MPI	Lung	rs1130741	15	0,117584	1,124776107	0,3676295
MPI	Brain Caudate basal ganglia	rs1130741	15	0,0481371	1,049314506	0,3679345
MPI	Cells EBV transformed lymphocytes	rs1130741	15	0,0548634	1,056396301	0,3709758
MPI	Brain Cortex	rs11630592	15	0,0507247	1,052033229	0,3939739
MPI	Testis	rs12904897	15	0,0710153	1,073597652	0,3966093
MPI	Brain Putamen basal ganglia	rs7497026	15	0,047341	1,04847948	0,4041523
MPI	Spleen	rs7497026	15	0,0507068	1,052014397	0,4046927
MPI	Adipose Subcutaneous	rs12906946	15	0,076107	1,079078029	0,544109
MPI	Brain Frontal Cortex BA9	rs6495124	15	0,0351244	1,035748548	0,5631397
MPI	Skin Sun Exposed Lower leg	rs6495126	15	0,0625045	1,064499249	0,5931276
MPI	Stomach	rs7497201	15	0,0458488	1,046916105	0,6154666

MPI	Artery Aorta	rs7497201	15	0,0720359	1,074693925	0,6156613
MPI	Adipose Visceral Omentum	rs11072517	15	0,0375699	1,038284571	0,654377
MPI	Muscle Skeletal	rs1531162	15	0,0572483	1,058918707	0,7707782
MPI	Brain Anterior cingulate cortex BA24	rs936227	15	0,00900015	1,009040773	0,8637381
MPI	Skin Not Sun Exposed Suprapubic	rs6939	15	0,00819012	1,008223751	0,9618699
NAB2	Blood	rs73338505	12	-0,451335	0,636777486	0,337568
NAT1	Brain Cerebellar Hemisphere	rs6994374	8	0,0593594	1,061156552	0,1494823
NAT1	Blood	rs6998240	8	-0,209392	0,811077231	0,2579933
NAT10	Adipose Subcutaneous	rs16925219	11	0,211522	1,235557148	0,203178
NAT10	Skin Sun Exposed Lower leg	rs2473932	11	0,247982	1,281436866	0,2070079
NAT10	Pancreas	rs16925219	11	0,175518	1,191863442	0,2086181
NAT10	Brain Cortex	rs2473932	11	0,104921	1,110622868	0,2087839
NAT10	Colon Transverse	rs2473932	11	0,206793	1,229727992	0,2100984
NAT10	Skin Not Sun Exposed Suprapubic	rs2473932	11	0,285138	1,329945548	0,2102163
NAT10	Stomach	rs2473932	11	0,174323	1,190440016	0,2115491
NAT10	Breast Mammary Tissue	rs2473932	11	0,240844	1,272322538	0,2118116
NAT10	Lung	rs2473932	11	0,387891	1,473869124	0,2128727
NAT10	Blood	rs2004272	11	0,132703	1,1419108	0,257839
NAT10	Esophagus Muscularis	rs112172055	11	0,16722	1,18201428	0,2624492
NAT10	Thyroid	rs112172055	11	0,167627	1,182495458	0,2628412
NAT10	Artery Tibial	rs79396490	11	0,139198	1,149351649	0,2635842
NAT10	Brain Cerebellum	rs112172055	11	0,0864932	1,090343953	0,2636476
NAT10	Cells Cultured fibroblasts	rs16925183	11	0,224616	1,251841917	0,2966838
NR3C1	Esophagus Mucosa	rs72804709	5	-0,134498	0,874154627	0,1047322
NR3C1	Blood	rs4986593	5	0,413669	1,512356454	0,2866217
P2RY8						
PAICS	Blood	rs1967559	4	0,168014	1,182953172	0,4442472
PAICS	Thyroid	rs17051687	4	0,0054542	1,005469101	0,9624669
PFKL	Skin Sun Exposed Lower leg	rs8134520	21	-0,123546	0,883780986	0,1007569
PFKL	Breast Mammary Tissue	rs34577649	21	-0,137538	0,871501232	0,1040949
PFKL	Testis	rs34577649	21	-0,230387	0,794226177	0,1093406
PFKL	Adipose Subcutaneous	rs2838545	21	-0,114953	0,891408039	0,1193634
PFKL	Skin Not Sun Exposed Suprapubic	rs2838545	21	-0,135524	0,873258204	0,1208606
PFKL	Adipose Visceral Omentum	rs2838545	21	-0,159489	0,852579346	0,1212257

PFKL	Esophagus Mucosa	rs2838545	21	-0,174164	0,8401591	0,1226927
PFKL	Brain Cortex	rs2838545	21	-0,0920684	0,912042764	0,1239509
PFKL	Artery Aorta	rs2838545	21	-0,159452	0,852610892	0,1240201
PFKL	Heart Left Ventricle	rs2838545	21	-0,231137	0,793630731	0,1261642
PFKL	Heart Atrial Appendage	rs2838545	21	-0,208922	0,811458527	0,1283831
PFKL	Lung	rs3788118	21	-0,110623	0,895276204	0,1295659
PFKL	Artery Tibial	rs3788118	21	-0,138104	0,871008102	0,1307285
PFKL	Esophagus Muscularis	rs3788118	21	-0,100875	0,904046032	0,1310146
PFKL	Thyroid	rs3788118	21	-0,176642	0,838079763	0,1320835
PFKL	Esophagus Gastroesophageal Junction	rs3788118	21	-0,11845	0,888296229	0,1351443
PFKL	Nerve Tibial	rs2070573	21	0,131855	1,140942871	0,3396325
PFKL	Whole blood	rs62220386	21	0,171993	1,187669519	0,4079131
PFKL	Brain Caudate basal ganglia	rs2838549	21	0,0439168	1,044895416	0,6781635
PFKL	Brain Spinal cord cervical c 1	rs2838549	21	0,0181246	1,018289847	0,6781706
PFKL	Blood	rs3761392	21	-0,0131243	0,986961448	0,9038466
PFKL	Colon Transverse	rs3761392	21	-0,0208118	0,979403271	0,9038623
PFKL	Stomach	rs3761392	21	-0,0182358	0,981929466	0,9038638
PFKL	Brain Cerebellum	rs3761392	21	-0,010211	0,989840955	0,9038654
PFKL	Muscle Skeletal	rs2071152	21	0,000523651	1,000523788	0,9947508
PGK1						
PHACTR1	Whole blood	rs1223563	6	0,196214	1,21678727	0,08428358
PHACTR1	Artery Tibial	rs9349379	6	0,081727	1,085159521	0,20703
PHACTR1	Artery Aorta	rs9349379	6	0,0981855	1,103167404	0,2100596
PHACTR1	Artery Coronary	rs9349379	6	0,0707019	1,073261239	0,2152369
PHACTR1	Blood	rs538190	6	-0,0652284	0,936853462	0,3043003
PHACTR1	Muscle Skeletal	rs6920793	6	-0,0882442	0,915537275	0,463759
PHACTR1	Heart Atrial Appendage	rs4236091	6	0,0470239	1,048147059	0,4973787
PHACTR1	Cells Cultured fibroblasts	rs417024	6	-0,0666029	0,935566641	0,5215523
PHACTR1	Skin Not Sun Exposed Suprapubic	rs417048	6	-0,0518973	0,949426368	0,6140429
PHACTR1	Adipose Subcutaneous	rs9381884	6	-0,0409551	0,959872227	0,6590387
PHACTR1	Esophagus Muscularis	rs34505804	6	-0,0405309	0,960279491	0,6929828
PHACTR1	Thyroid	rs17700321	6	-0,0474662	0,953642706	0,715023
PHACTR1	Skin Sun Exposed Lower leg	rs412787	6	-0,00721799	0,992807997	0,9491091
PHACTR1	Nerve Tibial	rs202041	6	-0,00201414	0,997987887	0,9734089

PIK3CG	Blood	rs42175	7	-0,470204	0,624874781	0,03249769
PIK3R1	Blood	rs4976156	5	0,290311	1,336843182	0,1095329
PRDM1						
PRL						
PRR7	Colon Sigmoid	rs2545795	5	-0,170469	0,843269231	0,02915791
PRR7	Cells Cultured fibroblasts	rs2545797	5	0,327075	1,386905491	0,04793613
PTP4A1	Blood	rs116293333	6	-0,256159	0,77401889	0,1278961
RBBP7						
RET	Lung	rs2435356	10	-0,106392	0,899072143	0,2133614
RET	Spleen	rs1864403	10	-0,0492022	0,951988618	0,2891454
RET	Cells Cultured fibroblasts	rs2251674	10	-0,0743539	0,928343095	0,308753
RET	Adipose Visceral Omentum	rs1864408	10	-0,0852467	0,918285715	0,3112117
RET	Nerve Tibial	rs12767776	10	-0,0904149	0,913552074	0,3296162
RET	Blood	rs2503841	10	-0,159781	0,852330429	0,4363914
RET	Skin Not Sun Exposed Suprapubic	rs2995411	10	-0,0566107	0,944961871	0,5026814
RET	Adipose Subcutaneous	rs2505990	10	-0,0358574	0,964777861	0,6489754
RET	Testis	rs2506013	10	-0,0208505	0,979365369	0,8292173
RPS13	Lung	rs75207092	11	-0,073694	0,928955911	0,7214679
RPS13	Artery Tibial	rs75207092	11	-0,128457	0,879451378	0,7217431
RPS13	Skin Sun Exposed Lower leg	rs11024218	11	-0,0321484	0,968362866	0,8870441
RPS13	Adipose Subcutaneous	rs11024218	11	-0,0339873	0,96658378	0,8870443
RPS13	Thyroid	rs68175505	11	-0,0300565	0,970390705	0,8879266
RPS13	Adipose Visceral Omentum	rs68175505	11	-0,0297509	0,970687302	0,8879268
RPS13	Nerve Tibial	rs68175505	11	-0,0334327	0,967119996	0,8879283
RPS13	Skin Not Sun Exposed Suprapubic	rs68175505	11	-0,0281147	0,97227684	0,8879284
RPS13	Breast Mammary Tissue	rs68175505	11	-0,0352405	0,965373216	0,8879322
RPS13	Esophagus Mucosa	rs68175505	11	-0,042429	0,958458514	0,8879357
RPS13	Colon Transverse	rs68175505	11	-0,0403374	0,960465323	0,887941
RPS13	Whole blood	rs68175505	11	-0,0740858	0,928592017	0,8879428
RPS13	Blood	rs68175505	11	-0,0788945	0,924137416	0,8879454
RPS13	Cells Cultured fibroblasts	rs68175505	11	-0,0646987	0,937349844	0,887948
RPS13	Artery Aorta	rs68175505	11	-0,0469624	0,954123272	0,8879529
RPS13	Testis	rs68175505	11	-0,032647	0,967880161	0,8879535
S100B	Adipose Subcutaneous	rs2839321	21	-0,122291	0,884890828	0,03979347

S100B	Adipose Visceral Omentum	rs2839321	21	-0,12288	0,884369781	0,04001727
S100B	Breast Mammary Tissue	rs2839321	21	-0,183496	0,832355205	0,04332719
S100B	Skin Sun Exposed Lower leg	rs2839321	21	-0,171032	0,842794604	0,04790935
S100B	Skin Not Sun Exposed Suprapubic	rs2839321	21	-0,205739	0,814045514	0,05205751
S100B	Blood	rs11701034	21	0,00960924	1,009655557	0,7608652
S100B	Whole blood	rs11701034	21	0,012406	1,012483274	0,7609351
SACM1L	Testis	rs9876474	3	-0,425507	0,653438408	0,04383781
SACM1L	Skin Not Sun Exposed Suprapubic	rs2673038	3	0,197425	1,218261692	0,07804062
SACM1L	Artery Tibial	rs2742386	3	-0,301222	0,739913494	0,08055997
SACM1L	Colon Transverse	rs6800556	3	-0,279993	0,755789032	0,08570917
SACM1L	Thyroid	rs2742462	3	-0,0997842	0,905032703	0,1052814
SACM1L	Adipose Subcutaneous	rs2742462	3	-0,283911	0,752833644	0,1170618
SACM1L	Skin Sun Exposed Lower leg	rs2742379	3	0,146231	1,157463531	0,1301744
SACM1L	Blood	rs2742433	3	0,0357016	1,036346555	0,5273973
SACM1L	Whole blood	rs2742433	3	0,139946	1,150211686	0,5289312
SLC18A2	Testis	rs4752051	10	-0,0884825	0,915319129	0,0892237
SLC18A2	Blood	rs181440	10	0,0694011	1,071866048	0,5883812
SLC18A2	Stomach	rs363225	10	-0,0140936	0,98600525	0,862716
SLC25A1	Skin Not Sun Exposed Suprapubic	rs885978	22	0,0951884	1,09986605	0,3665174
SLC25A1	Skin Sun Exposed Lower leg	rs2518840	22	0,113794	1,120521274	0,4020303
SLC25A1	Pancreas	rs698423	22	0,0628156	1,064830466	0,4586615
SLC25A1	Cells Cultured fibroblasts	rs2073740	22	0,0410918	1,041947752	0,5133435
SLC25A1	Artery Aorta	rs2073740	22	0,0638551	1,065937933	0,5149864
SLC25A1	Nerve Tibial	rs9604934	22	0,0519119	1,053282944	0,5287771
SLC25A1	Artery Tibial	rs9604934	22	0,0735728	1,076346892	0,5289525
SLC25A1	Lung	rs9604934	22	0,0584085	1,060147978	0,5290036
SLC25A1	Esophagus Gastroesophageal Junction	rs9604934	22	0,0665359	1,068799333	0,5301657
SLC25A1	Blood	rs1018764	22	0,0485909	1,049790793	0,5847011
SLC25A1	Heart Left Ventricle	rs1018764	22	0,028688	1,029103464	0,5851939
SLC25A1	Whole blood	rs1018764	22	0,0786379	1,081812527	0,5857907
SLC25A1	Esophagus Mucosa	rs9605957	22	0,0512816	1,052619269	0,6100812
SLC25A1	Colon Sigmoid	rs9605957	22	0,0449619	1,045988007	0,6103038
SLC25A1	Esophagus Muscularis	rs1091013	22	0,0460646	1,047142054	0,6473036
SLC6A2	Testis	rs11645905	16	0,0986276	1,103655222	0,2900234

SLC6A2	Esophagus Gastroesophageal Junction	rs55830311	16	0,07541	1,078326174	0,4717582
SLC6A2	Skin Not Sun Exposed Suprapubic	rs17841327	16	-0,0122711	0,987803883	0,6943945
SLC6A2	Skin Sun Exposed Lower leg	rs17841327	16	-0,0133644	0,986724507	0,6943981
SLC6A2	Adrenal Gland	rs17841327	16	-0,0227125	0,977543487	0,6946241
SLC6A2	Esophagus Muscularis	rs36030	16	-0,0225894	0,97766383	0,7376576
SLC6A3	Lung	rs2550946	5	0,0128614	1,012944464	0,7112296
SLC6A3	Thyroid	rs2550946	5	0,0194681	1,019658839	0,7112745
SLC6A3	Skin Not Sun Exposed Suprapubic	rs2550946	5	0,0235226	1,023801438	0,7114512
SLC6A3	Ovary	rs2550946	5	0,0156993	1,015823181	0,7115431
SLC6A3	Adipose Visceral Omentum	rs2550946	5	0,0388888	1,039654868	0,7115557
SLC6A3	Skin Sun Exposed Lower leg	rs2937650	5	-0,00169298	0,998308452	0,9842947
SLC6A4	Artery Aorta	rs34622227	17	0,0204257	1,020635732	0,7254982
SLC6A4	Heart Atrial Appendage	rs12939390	17	0,025708	1,026041301	0,7288093
SLC6A4	Esophagus Muscularis	rs12947654	17	0,0209369	1,021157615	0,7391705
SMAD2	Blood	rs1792681	18	0,279597	1,322596698	0,2599843
SMAD2	Cells Cultured fibroblasts	rs8098413	18	-0,0420569	0,958815222	0,5564117
SMAD2	Artery Tibial	rs8098413	18	-0,0525748	0,948783349	0,5566194
SMAD2	Muscle Skeletal	rs8098413	18	-0,0612137	0,940622207	0,5568508
SMAD2	Adipose Subcutaneous	rs8098413	18	-0,0717557	0,930758252	0,5569092
SMAD2	Esophagus Muscularis	rs8098413	18	-0,0756069	0,92718061	0,5572814
SMAD2	Nerve Tibial	rs8098413	18	-0,0751137	0,927638008	0,5573167
SMAD2	Colon Sigmoid	rs8098413	18	-0,0670575	0,935141429	0,5576325
SMAD2	Artery Aorta	rs8098413	18	-0,0726392	0,929936291	0,5577984
SMU1	Adipose Visceral Omentum	rs13289835	9	0,164627	1,178953287	0,4085934
SMU1	Heart Atrial Appendage	rs13289835	9	0,113169	1,119821167	0,4089573
SMU1	Blood	rs10758180	9	0,0773393	1,080408597	0,4553944
SMU1	Whole blood	rs2274767	9	0,0964975	1,101306828	0,4595993
SMU1	Muscle Skeletal	rs2274767	9	0,073854	1,076649603	0,4600504
SMU1	Testis	rs2274767	9	0,0664776	1,068737024	0,4603196
SMU1	Nerve Tibial	rs2274767	9	0,0826301	1,086139971	0,4606422
SMU1	Esophagus Mucosa	rs2274767	9	0,105213	1,110947217	0,4608322
SMU1	Thyroid	rs2274767	9	0,131095	1,140076083	0,4609031
SMU1	Breast Mammary Tissue	rs10971316	9	0,0662193	1,068461005	0,4756407
SMU1	Skin Not Sun Exposed Suprapubic	rs10971316	9	0,105819	1,111620655	0,4757958

SMU1	Artery Aorta	rs10971316	9	0,0842187	1,087866784	0,4762238
SMU1	Artery Tibial	rs7872744	9	0,0615612	1,06349558	0,4775028
SMU1	Esophagus Gastroesophageal Junction	rs7872744	9	0,0974636	1,102371315	0,4796385
SMU1	Adipose Subcutaneous	rs7043066	9	0,0656979	1,067904055	0,4862354
SMU1	Cells Cultured fibroblasts	rs1537040	9	0,0631228	1,065157633	0,529207
SMU1	Skin Sun Exposed Lower leg	rs1537040	9	0,0908393	1,09509301	0,52958
SMU1	Lung	rs76124374	9	-0,0397954	0,960986037	0,8313544
SMU1	Colon Transverse	rs76124374	9	-0,0337671	0,966796645	0,8313641
SMU1	Stomach	rs76124374	9	-0,0309719	0,969502816	0,8313652
SMU1	Esophagus Muscularis	rs76124374	9	-0,042119	0,958755682	0,8313697
SPP1	Testis	rs11938899	4	-0,0672551	0,934956663	0,01644859
SPP1	Spleen	rs12644436	4	-0,0921783	0,911942536	0,03119282
SPP1	Whole blood	rs1471400	4	-0,114114	0,892156244	0,1358768
SPP1	Thyroid	rs17013569	4	-0,101075	0,90386524	0,1567483
SPP1	Artery Tibial	rs1471401	4	-0,0923457	0,911789889	0,1923875
SPP1	Esophagus Muscularis	rs28357094	4	0,0794578	1,082699869	0,202545
SPP1	Esophagus Gastroesophageal Junction	rs28357094	4	0,064169	1,066272584	0,2030866
SPP1	Nerve Tibial	rs1471403	4	-0,071123	0,931347329	0,2055039
SPP1	Adipose Subcutaneous	rs1471403	4	-0,105343	0,900015764	0,209196
SPP1	Adipose Visceral Omentum	rs5024096	4	-0,0521825	0,94915563	0,4350662
SPP1	Blood	rs2853749	4	0,0176056	1,017761492	0,7134333
SPP1	Esophagus Mucosa	rs2853749	4	0,0189656	1,019146589	0,7137593
SPP1	Pancreas	rs1381949	4	-0,00472207	0,995289061	0,937407
SUPT16H						
TFF2	Thyroid	rs178740	21	0,0344557	1,035056174	0,4810781
TLR5	Blood	rs851180	1	0,033411	1,033975416	0,8033879
UMODL1	Pituitary	rs11701944	21	0,0492979	1,050533258	0,1934567
UMODL1	Blood	rs55787571	21	0,261039	1,298278297	0,2403712
UMODL1	Lung	rs55999015	21	0,029866	1,030316462	0,2468694
UMODL1	Thyroid	rs35520919	21	0,030804	1,031283353	0,2633748
UMODL1	Spleen	rs35520919	21	0,0260609	1,026403455	0,2648827
UMODL1	Small Intestine Terminal Ileum	rs66994219	21	0,0629927	1,065019065	0,3495447
UMODL1	Testis	rs220287	21	-0,0277351	0,972645987	0,5193931
USP32						

WARS2	Blood	rs2148150	1	0,0315237	1,032025834	0,4402205
WARS2	Skin Sun Exposed Lower leg	rs12086	1	0,0081964	1,008230082	0,7463216
WARS2	Thyroid	rs12086	1	0,00816014	1,008193525	0,7463265
WARS2	Nerve Tibial	rs12086	1	0,00914451	1,009186449	0,7463304
WARS2	Lung	rs12086	1	0,010006	1,010056227	0,7463385
WARS2	Esophagus Mucosa	rs12086	1	0,0113782	1,011443178	0,74634
WARS2	Breast Mammary Tissue	rs12086	1	0,0089917	1,009032247	0,7463493
WARS2	Adipose Visceral Omentum	rs12086	1	0,0118839	1,011954794	0,7463508
WARS2	Colon Transverse	rs12086	1	0,0113843	1,011449348	0,7463519
WARS2	Stomach	rs12086	1	0,0137052	1,013799547	0,7463637
WARS2	Pancreas	rs12086	1	0,0125638	1,012643056	0,7463831
WARS2	Heart Left Ventricle	rs12086	1	0,0175299	1,01768445	0,7463891
WARS2	Prostate	rs12086	1	0,0080576	1,00809015	0,7464009
WARS2	Minor Salivary Gland	rs12086	1	0,00804435	1,008076793	0,7464141
WARS2	Spleen	rs12086	1	0,00932418	1,009367786	0,7464146
WARS2	Ovary	rs12086	1	0,0111465	1,011208854	0,7464982
WARS2	Cells EBV transformed lymphocytes	rs12086	1	0,00984416	1,009892773	0,7464989
WARS2	Whole blood	rs2765543	1	0,0303601	1,030825667	0,7881654
WARS2	Brain Cerebellum	rs2794315	1	0,00436954	1,0043791	0,8407526
WARS2	Pituitary	rs2794315	1	0,00552471	1,005539999	0,8407588
WARS2	Brain Cortex	rs2794315	1	0,00456815	1,0045786	0,8407602
WARS2	Brain Hypothalamus	rs2794315	1	0,00595773	1,005975513	0,8407725
WARS2	Testis	rs78641692	1	-0,00876249	0,991275789	0,8543107
WARS2	Muscle Skeletal	rs2645291	1	0,00452106	1,004531295	0,8896457
WARS2	Skin Not Sun Exposed Suprapubic	rs2645291	1	0,00390754	1,003915184	0,8896458
WARS2	Adrenal Gland	rs2645291	1	0,00466123	1,00467211	0,8896513
WARS2	Brain Cerebellar Hemisphere	rs2645291	1	0,00311987	1,003124742	0,8896521
WARS2	Brain Nucleus accumbens basal ganglia	rs2645291	1	0,0047399	1,004751151	0,889655
WARS2	Brain Putamen basal ganglia	rs2645291	1	0,00357669	1,003583094	0,8896551
WARS2	Small Intestine Terminal Ileum	rs2645291	1	0,0053058	1,005319901	0,8896588
WARS2	Liver	rs2645291	1	0,00730341	1,007330145	0,8896651
WARS2	Brain Substantia nigra	rs2645291	1	0,00414146	1,004150048	0,8896661
WARS2	Vagina	rs2645291	1	0,00493778	1,004949991	0,889671
WARS2	Adipose Subcutaneous	rs2645303	1	0,00348171	1,003487778	0,9085793

WARS2	Cells Cultured fibroblasts	rs2645303	1	0,00490659	1,004918647	0,9085798
WARS2	Artery Tibial	rs2645303	1	0,0040396	1,00404777	0,9085798
WARS2	Artery Aorta	rs2645303	1	0,00417638	1,004185113	0,9085815
WARS2	Heart Atrial Appendage	rs2645303	1	0,00364549	1,003652143	0,9085815
WARS2	Esophagus Muscularis	rs2645303	1	0,00481011	1,004821697	0,9085815
WARS2	Esophagus Gastroesophageal Junction	rs2645303	1	0,00455965	1,004570061	0,9085816
WARS2	Brain Frontal Cortex BA9	rs2645303	1	0,00259364	1,002597006	0,9085825
WARS2	Colon Sigmoid	rs2645303	1	0,00430997	1,004319271	0,9085827
WARS2	Artery Coronary	rs2645303	1	0,00422221	1,004231136	0,9085835
WARS2	Brain Caudate basal ganglia	rs2645303	1	0,00352137	1,003527577	0,9085837
WARS2	Brain Anterior cingulate cortex BA24	rs2645303	1	0,00307364	1,003078368	0,9085843
WARS2	Brain Hippocampus	rs2645303	1	0,00342636	1,003432237	0,9085851
WARS2	Brain Spinal cord cervical c 1	rs2645303	1	0,00289113	1,002895313	0,9085873
WARS2	Adipose Visceral Omentum	rs2645303	1	0,00322765	1,003232864	0,9085875
WARS2	Brain Amygdala	rs2645303	1	0,00322765	1,003232864	0,9085875
ZNF595						
ZPBP2	Testis	rs12232497	17	-0,101612	0,903379995	0,3218243

Supplementary Table S6 Summary-data-based Mendelian randomization results of *HYKK* on IA, smoking behavior, CRP level and 5-hydroxylysine level risks

Gene	Outcome	Top SNP	TopSNP CHR	topSNP BP	A1	A2	Freq	GWAS beta	GWAS se	GWAS p-value	eQTL beta	eQTL se
HYKK	SmkAge	rs931794	15	78826180	G	A	0,380368	0,00607	0,00315	0,111	-0,0547641	0,00920869
HYKK	CigDay	rs931794	15	78826180	G	A	0,380368	0,014	0,00256	1,04E-08	-0,0547641	0,00920869
HYKK	SmkCes	rs931794	15	78826180	G	A	0,380368	0,0897	0,00293	2,19E-205	-0,0547641	0,00920869
HYKK	SmkInt	rs931794	15	78826180	G	A	0,380368	-0,00441	0,00186	0,00962	-0,0547641	0,00920869
HYKK	ALL IA	rs931794	15	78826180	G	A	0,380368	0,1138	0,0218	1,866E-07	-0,0547641	0,00920869
HYKK	Only uIA	rs931794	15	78826180	G	A	0,380368	0,0994	0,0406	0,01443	-0,0547641	0,00920869
HYKK	Only SAH	rs931794	15	78826180	G	A	0,380368	0,1157	0,0251	0,000004182	-0,0547641	0,00920869
HYKK	CRP level	rs931794	15	78826180	G	A	0,380368	0,0129519	0,00212943	1,18439E-09	-0,0547641	0,00920869
HYKK	5-hydroxylysine level	rs931794	15	78826180	G	A	0,380368	-0,0384851	0,0160682	0,0166154	-0,0547641	0,00920869

eQTL p-value	SMR Beta	SMR se	SMR p-value	HEIDI p-value	HEIDI nsnp
2,731E-09	-0,110839	0,0604637	0,06678003	0,05601998	20
2,731E-09	-0,255642	0,0635062	5,68634E-05	0,4637417	20
2,731E-09	-1,63793	0,28057	5,28711E-09	0,2337858	20
2,731E-09	0,0805272	0,0365636	0,02763798	0,4172888	20
2,731E-09	-2,078	0,529675	8,73913E-05	0,7573754	20
2,731E-09	-1,81506	0,801728	0,02357826	0,7399377	20
2,731E-09	-2,1127	0,579889	0,000269188	0,7620882	20
2,731E-09	-0,236504	0,055619	2,11674E-05	0,3520409	20
2,731E-09	0,702743	0,316309	0,02630384	2,87089E-06	20

Supplementary Table S7 Colocalization analysis of the *HYKK* locus between gene expressi

Trait 1	Trait 2	Most likely SNP	PP.H4
5-hydroxylysine level	all IA	-	-
5-hydroxylysine level	only SAH	-	-
5-hydroxylysine level	only uIA	-	-
CigDay	all IA	rs10519203	0,999
CigDay	only SAH	rs10519203	0,995
CigDay	only uIA	rs10519203	0,818
SmkInt	all IA	rs10519203	0,03
SmkInt	only SAH	rs10519203	0,017
SmkInt	only uIA	rs12911436	0,007
CRP level	all IA	rs10519203	0,95
CRP level	only SAH	rs11852372	0,97
CRP level	only uIA	rs11852372	0,366
HYKK expression in blood	all IA	rs10519203	0,989
HYKK expression in blood	only SAH	rs10519203	0,983
HYKK expression in blood	only uIA	rs10519203	0,506
HYKK expression in blood	CigDay	rs10519203	0,989
HYKK expression in blood	SmkInt	-	-
HYKK expression in blood	CRP level	rs11852372	0,986
HYKK expression in blood	5-hydroxylysine level	-	-

Supplementary Table S8 Mendelian randomization results of CRP level and 5-hydroxylysine level on IA risk

exposure	outcome	method	nsnp	beta	se	p-value	beta lower 95%CI
CRP level	All IA	MR Egger	214	0,154646031	0,155601947	0,321425663	-0,150333785
CRP level	All IA	Weighted median	214	0,097843819	0,134605122	0,467290149	-0,16598222
CRP level	All IA	Inverse variance weighted	214	0,100030659	0,088260121	0,257062307	-0,072959179
CRP level	All IA	Simple mode	214	-0,367842058	0,264542168	0,165832612	-0,886344707
CRP level	All IA	Weighted mode	214	0,061106038	0,13537489	0,652172163	-0,204228747
CRP level	Only uIA	MR Egger	214	0,116367102	0,237130603	0,62412498	-0,34840888
CRP level	Only uIA	Weighted median	214	0,192043584	0,223136718	0,389428448	-0,245304382
CRP level	Only uIA	Inverse variance weighted	214	0,053770094	0,135207419	0,690861679	-0,211236448
CRP level	Only uIA	Simple mode	214	-0,592155191	0,529162736	0,264382703	-1,629314153
CRP level	Only uIA	Weighted mode	214	0,265161843	0,24184397	0,274134385	-0,208852338
CRP level	Only SAH	MR Egger	214	0,202994492	0,172107215	0,239534844	-0,13433565
CRP level	Only SAH	Weighted median	214	0,142623175	0,15601262	0,36062376	-0,16316156
CRP level	Only SAH	Inverse variance weighted	214	0,107024114	0,097385542	0,271779699	-0,083851549
CRP level	Only SAH	Simple mode	214	-0,502887699	0,338543939	0,138905048	-1,166433819
CRP level	Only SAH	Weighted mode	214	0,116329864	0,136862622	0,396293143	-0,151920876
5-hydroxylysine level	All IA	Wald ratio	1	0,139300318	0,066366405	0,035820574	0,009222165
5-hydroxylysine level	Only SAH	Wald ratio	1	0,160731137	0,076563326	0,035788455	0,010667017
5-hydroxylysine level	Only uIA	Wald ratio	1	0,064638113	0,124437009	0,603450845	-0,179258425

beta upper 95%CI	or	or lower 95%CI	or upper 95%CI
0,459625848	1,16724472	0,860420733	1,583481411
0,361669857	1,102790536	0,847061295	1,435724869
0,273020497	1,105204802	0,929638778	1,313927176
0,15066059	0,692226504	0,412159566	1,162601993
0,326440823	1,063011628	0,815275858	1,386026226
0,581143085	1,123408202	0,705810225	1,788081192
0,629391551	1,211723328	0,782466333	1,876468496
0,318776636	1,055241968	0,80958262	1,375444066
0,445003771	0,55313389	0,196063998	1,56049608
0,739176023	1,303641944	0,811515057	2,094209224
0,540324635	1,225065721	0,874296558	1,716564028
0,44840791	1,153295129	0,849453939	1,565817278
0,297899776	1,112961092	0,919567757	1,347026777
0,16065842	0,604781708	0,311475742	1,174283789
0,384580604	1,123366369	0,85905625	1,4689981
0,269378472	1,149469255	1,00926482	1,309150524
0,310795256	1,174369181	1,010724112	1,364509818
0,308534651	1,066772903	0,835889857	1,361428683

Supplementary Table S9 Mendelian randomization results of HYKK and CigDay on IA risk using rs7164594 only as instruments

Exposure	Outcome	Method	Nsnp	Beta	SE	p-value	beta lower 95%CI	beta upper 95%CI
HYKK only rs7164594	All IA	Wald ratio	1	-2,078003656	0,398070999	1,78746E-07	-2,858222814	-1,297784498
HYKK only rs7164594	Only SAH	Wald ratio	1	-2,945110135	0,783361083	0,000170198	-4,480497857	-1,409722412
HYKK only rs7164594	Only uIA	Wald ratio	1	-2,172666976	1,258290799	0,084224947	-4,638916942	0,29358299
CigDay only rs7164594	All IA	Wald ratio	1	1,405874499	0,332443258	2,3482E-05	0,754285714	2,057463284
CigDay only rs7164594	Only SAH	Wald ratio	1	1,44058745	0,38317757	0,000170198	0,689559413	2,191615487
CigDay only rs7164594	Only uIA	Wald ratio	1	1,062750334	0,615487316	0,084224947	-0,143604806	2,269105474

or	or lower 95%CI	or upper 95%CI
0,125179865	0,057370628	0,273136257
0,052596267	0,011327772	0,244211064
0,113873514	0,009668163	1,341224483
4,079092346	2,126092343	7,826092039
4,223175993	1,992837321	8,949659504
2,8943204	0,866230009	9,67074621

Fig 4. Associations between varenicline, the HYKK gene, smoking behavior, and intracranial aneurysm.

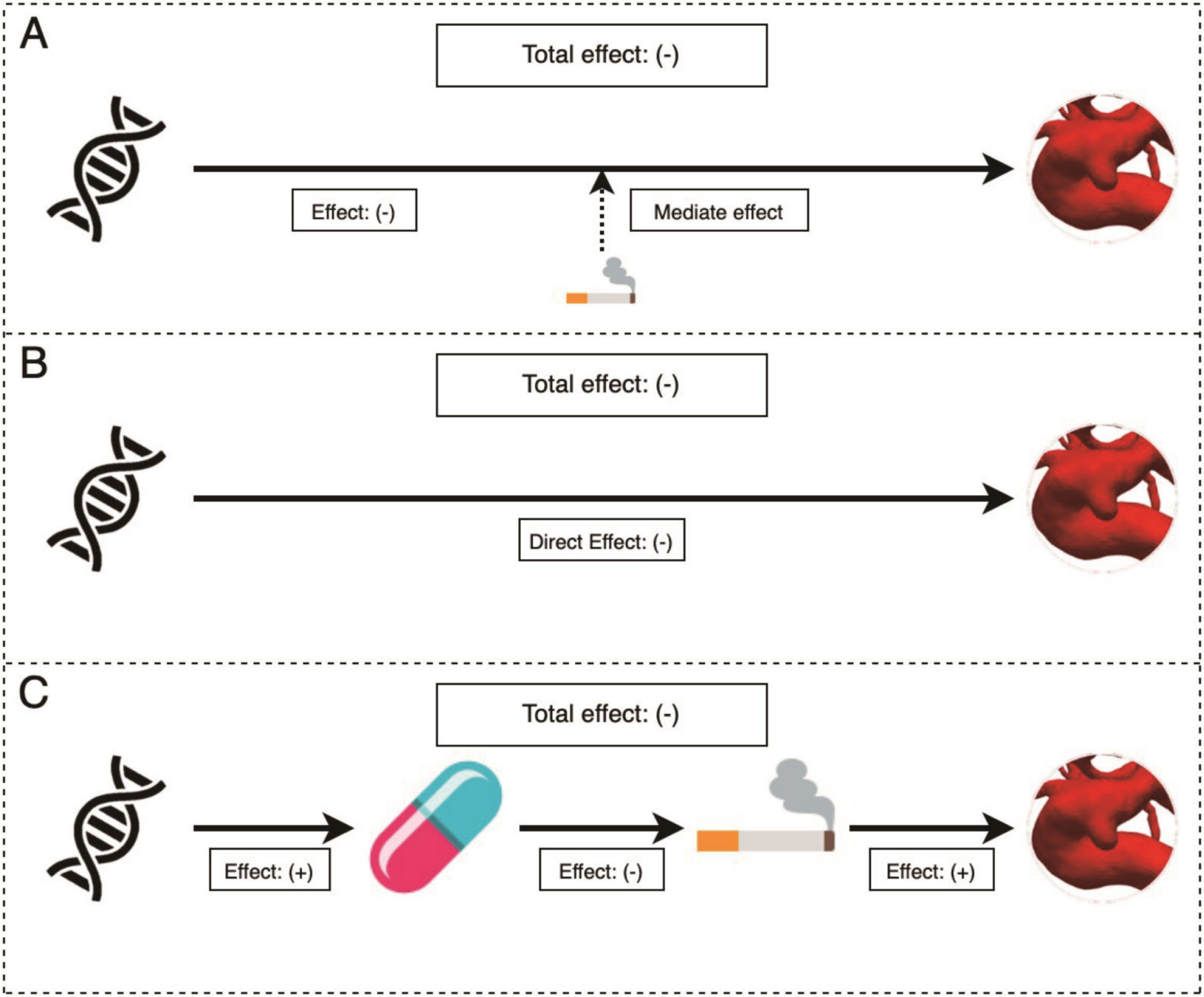

STROBE Statement—checklist of items that should be included in reports of observational studies

	Item No	Recommendation
**Title and abstract**	1	(*a*) Indicate the study's design with a commonly used term in the title or the abstract
		(*b*) Provide in the abstract an informative and balanced summary of what was done and what was found
**Introduction**		
Background/rationale	2	Explain the scientific background and rationale for the investigation being reported
Objectives	3	State specific objectives, including any prespecified hypotheses
**Methods**		
Study design	4	Present key elements of study design early in the paper
Setting	5	Describe the setting, locations, and relevant dates, including periods of recruitment, exposure, follow-up, and data collection
Participants	6	(*a*) *Cohort study*—Give the eligibility criteria, and the sources and methods of selection of participants. Describe methods of follow-up<br>*Case-control study*—Give the eligibility criteria, and the sources and methods of case ascertainment and control selection. Give the rationale for the choice of cases and controls<br>*Cross-sectional study*—Give the eligibility criteria, and the sources and methods of selection of participants
		(*b*) *Cohort study*—For matched studies, give matching criteria and number of exposed and unexposed<br>*Case-control study*—For matched studies, give matching criteria and the number of controls per case
Variables	7	Clearly define all outcomes, exposures, predictors, potential confounders, and effect modifiers. Give diagnostic criteria, if applicable
Data sources/ measurement	8*	For each variable of interest, give sources of data and details of methods of assessment (measurement). Describe comparability of assessment methods if there is more than one group
Bias	9	Describe any efforts to address potential sources of bias
Study size	10	Explain how the study size was arrived at
Quantitative variables	11	Explain how quantitative variables were handled in the analyses. If applicable, describe which groupings were chosen and why
Statistical methods	12	(*a*) Describe all statistical methods, including those used to control for confounding
		(*b*) Describe any methods used to examine subgroups and interactions
		(*c*) Explain how missing data were addressed
		(*d*) *Cohort study*—If applicable, explain how loss to follow-up was addressed<br>*Case-control study*—If applicable, explain how matching of cases and controls was addressed<br>*Cross-sectional study*—If applicable, describe analytical methods taking account of sampling strategy
		(*e*) Describe any sensitivity analyses

Continued on next page

**Results**		
Participants	13*	(a) Report numbers of individuals at each stage of study—eg numbers potentially eligible, examined for eligibility, confirmed eligible, included in the study, completing follow-up, and analysed
		(b) Give reasons for non-participation at each stage
		(c) Consider use of a flow diagram
Descriptive data	14*	(a) Give characteristics of study participants (eg demographic, clinical, social) and information on exposures and potential confounders
		(b) Indicate number of participants with missing data for each variable of interest
		(c) *Cohort study*—Summarise follow-up time (eg, average and total amount)
Outcome data	15*	*Cohort study*—Report numbers of outcome events or summary measures over time
		Case-control study—Report numbers in each exposure category, or summary measures of exposure
		Cross-sectional study—Report numbers of outcome events or summary measures
Main results	16	(*a*) Give unadjusted estimates and, if applicable, confounder-adjusted estimates and their precision (eg, 95% confidence interval). Make clear which confounders were adjusted for and why they were included
		(*b*) Report category boundaries when continuous variables were categorized
		(*c*) If relevant, consider translating estimates of relative risk into absolute risk for a meaningful time period
Other analyses	17	Report other analyses done—eg analyses of subgroups and interactions, and sensitivity analyses
**Discussion**		
Key results	18	Summarise key results with reference to study objectives
Limitations	19	Discuss limitations of the study, taking into account sources of potential bias or imprecision. Discuss both direction and magnitude of any potential bias
Interpretation	20	Give a cautious overall interpretation of results considering objectives, limitations, multiplicity of analyses, results from similar studies, and other relevant evidence
Generalisability	21	Discuss the generalisability (external validity) of the study results
**Other information**		
Funding	22	Give the source of funding and the role of the funders for the present study and, if applicable, for the original study on which the present article is based

*Give information separately for cases and controls in case-control studies and, if applicable, for exposed and unexposed groups in cohort and cross-sectional studies.

Note: An Explanation and Elaboration article discusses each checklist item and gives methodological background and published examples of transparent reporting. The STROBE checklist is best used in conjunction with this article (freely available on the Web sites of PLoS Medicine at http://www.plosmedicine.org/, Annals of Internal Medicine at http://www.annals.org/, and Epidemiology at http://www.epidem.com/). Information on the STROBE Initiative is available at www.strobe-statement.org.

©2024 Liang X. et al.
